# Proteomic Analysis of Vitreous Humor in Retinal Vein Occlusion

**DOI:** 10.1371/journal.pone.0158001

**Published:** 2016-06-30

**Authors:** Michael Reich, Ivanka Dacheva, Matthias Nobl, Justyna Siwy, Joost P. Schanstra, William Mullen, Frank H. J. Koch, Jürgen Kopitz, Florian T. A. Kretz, Gerd U. Auffarth, Michael J. Koss

**Affiliations:** 1 Eye Center, Albert-Ludwigs-University Freiburg, Freiburg, Germany; 2 Department of Ophthalmology, University of Heidelberg, Heidelberg, Germany; 3 Mosaiques Diagnostics GmbH, Hannover, Germany; 4 Institut National de la Santé et de la Recherche Médicale (INSERM), U1048, Institut of Cardiovascular and Metabolic Disease, Toulouse, France; 5 Université Toulouse III Paul-Sabatier, Toulouse, France; 6 BHF Glasgow Cardiovascular Research Centre, University of Glasgow, Glasgow, United Kingdom; 7 Department of Ophthalmology, University of Frankfurt, Frankfurt am Main, Germany; 8 Department of Pathology, University of Heidelberg, Heidelberg, Germany; 9 Department of Ophthalmology, University of Southern California, Los Angeles, California, United States of America; Pacific Northwest National Laboratory, UNITED STATES

## Abstract

**Purpose:**

To analyze the protein profile of human vitreous of untreated patients with retinal vein occlusion (RVO).

**Methods:**

Sixty-eight vitreous humor (VH) samples (44 from patients with treatment naïve RVO, 24 controls with idiopathic floaters) were analyzed in this clinical-experimental study using capillary electrophoresis coupled to mass spectrometer and tandem mass spectrometry. To define potential candidate protein markers of RVO, proteomic analysis was performed on RVO patients (n = 30) and compared with controls (n = 16). To determine validity of potential biomarker candidates in RVO, receiver operating characteristic (ROC) was performed by using proteome data of independent RVO (n = 14) and control samples (n = 8).

**Results:**

Ninety-four different proteins (736 tryptic peptides) could be identified. Sixteen proteins were found to be significant when comparing RVO and control samples (*P* = 1.43E-05 to 4.48E-02). Five proteins (Clusterin, Complement C3, Ig lambda-like polypeptide 5 (IGLL5), Opticin and Vitronectin), remained significant after using correction for multiple testing. These five proteins were also detected significant when comparing subgroups of RVO (central RVO, hemi-central RVO, branch RVO) to controls. Using independent samples ROC-Area under the curve was determined proving the validity of the results: Clusterin 0.884, Complement C3 0.955, IGLL5 1.000, Opticin 0.741, Vitronectin 0.786. In addition, validation through ELISA measurements was performed.

**Conclusion:**

The results of the study reveal that the proteomic composition of VH differed significantly between the patients with RVO and the controls. The proteins identified may serve as potential biomarkers for pathogenesis induced by RVO.

## Introduction

Retinal vein occlusion (RVO) is the second most common cause of vision loss in older patients due to retinal vascular disease after diabetic retinopathy [1,2]. Despite major achievements in diagnosis and treatment perspectives based on spectral OCT, there is still limited understanding of the pathophysiology of RVO [3–6]. A number of cytokines including vascular endothelial growth factor (VEGF) and interleukin-6 (IL-6) have been shown to be associated to RVO. However these cytokines display high interindividual variations and their concentrations often do not correspond to the associated clinical RVO phenotype [7,8]. Even in non diseased eyes, reproducible vitreous protein profiling is quite complicated as the aging vitreous incorporates different stages of liquefaction and syneresis, which is aggravated after treatment with intravitreal injections [9]. Therefore, many aspects of the molecular mechanism of RVO remain poorly described.

Proteomics is a promising approach allowing the analysis of the total protein content of a sample and in combination with robust statistical analyses can lead to the identification of proteins associated to specific diseases in ophthalmology [10–13].

In the context of RVO the current proteomic data are incomplete. To the best of our knowledge, only one proteome study in RVO exists. Yao *et al*. 2013 described the proteome of aqueous humor from patients with branch RVO (BRVO; n = 6) compared to controls (n = 6) [14].

Therefore, to potentially improve upon the insight in the pathophysiology of RVO we performed high-resolution proteome analysis of vitreous humor (VH) of a high number of patients with RVO (n = 44).

## Materials and Methods

### Study design

This was a clinical-experimental study. Samples were collected after the approval from local institutional review board (351/12) of the Goethe University Frankfurt am Main (Germany) in accordance with the European Guidelines for Good Clinical Practice and the Declaration of Helsinki. Written informed consent was obtained from each patient before the start of therapy (linked to the source file of each patient, which was approved by a local ethics committee).

### Patient characterization

In our retrospective case series we analyzed a total of 68 undiluted samples from previously untreated (any intravitreal drug application) patients for VH proteome identification and quantitative verification; 44 from RVO patients and 24 randomly chosen controls with idiopathic floaters. All patients consulted our Department of Ophthalmology between April and August 2012. Patients suffering neovascular complications such as rubeosis irids, vascularisations of iridocorneal chamber, neovascularisations of the papilla or in the retina perimeter were considered ineligible for this study as well as patients with vitreo-macular traction or patients previously treated with intravitreal anti-VEGF, intraocular steroids, coagulation with photo laser, vitrectomy or previous intraocular operations, such as cataract operation at the included or not included eye in the last six months. Patients with diabetic retinopathy, uveitis, glaucoma or other compromising ocular conditions were also excluded. For all participants personal data, such as gender and patient-age, were collected as well as information regarding type of RVO (central-RVO (CRVO), hemi-central RVO (H-CRVO), BRVO), lens status, averaged and central retinal thickness, posterior attachment of the vitreous body and presence of retinal cysts with spectral domain OCT (SD-OCT; Topcon 200, Tokyo, Japan).

### Tryptic digestion of vitreous

Sampling of VH, sample preparation and analysis was conducted as described in Koss *et al*. 2014 [12]. In short: 10 μL of the thawed samples were diluted 1:10 with 0.1% SDS, 20 mM DTT, and 0.1 M Tris-HCl (pH = 7.6). Samples were sonicated at room temperature for 30 minutes. Afterwards, the samples were denaturated at 95°C for 3 min and then were incubated for 30 min at room temperature in the absence of light with 0.05 M Iodoacetamide. Eight M urea, 0.2 M Tris-HCl and 50 mM ammonium bicarbonate buffer solution was added and the samples were applied to NAP-5 columns equilibrated in 50 mM ammonium bicarbonate buffer solution. Trypsin solution was added to the desalted samples. Trypsin digestion was carried out overnight at a temperature of 37°C. Subsequently, the samples were lyophilized. Afterwards the samples were stored at 4°C and were resuspended with 15 μl distilled water shortly before mass spectrometry analysis.

### CE-MS analysis

Capillary electrophoresis coupled to mass spectrometer (CE-MS) analysis was carried out as described by Theodorescu *et al*. 2006 [15]. A P/ACE MDQ capillary electrophoresis system (Beckman Coulter, Brea, CA) being linked online to a micro-TOF MS (Bruker Daltonik, Leipzig, Germany) was used. The sprayer (Agilent Technologies, Santa Clara, CA) interfacing the CE and MS was grounded. 256 nl of the sample was injected hydrodynamically on an untreated silica capillary (New Objective, Woburn, USA, 90 cm x 50 μm). A solution of 20% acetonitrile (Sigma-Aldrich, Taufkirchen, Germany) in HPLC-grade water (Roth, Karlsruhe, Germany) supplemented with 0.94% formic acid (Sigma-Aldrich) was used as running buffer. Interface potential was adjusted to -4.5 kV. The capillary temperature was held at 35°C. Mass spectra were recorded for three seconds with signals at an m/z range between 350 and 3000. The detection limit of the TOF-Analyzer was 1 fmol [15].

### Data processing of CE-MS analysis

Analysis of raw CE-MS data was carried out via MosaiquesVisu version 2.1.0 (mosaigues diagnostics GmbH, Hannover, Germany) which uses isotope identification and conjugated mass detection for mass deconvolution [16]. Only signals observed in a minimum of 3 consecutive spectra with a signal-to-noise (SNR) ratio of at least 4 were considered. The observed minimal signal intensity with SNR>4 was 1.2. The software automatically eliminates all signals that can be detected only as singly charged species. The software employs a probabilistic clustering algorithm and uses both, isotopic distribution and conjugated masses for charge-state determination of peptides/proteins. The resulting peak list characterizes each polypeptide by its molecular mass, CE-migration time, and ion signal intensity (amplitude) value. Mass spectral ion peaks from the same molecule at different charge states were deconvoluted and summarized into a single mass. To minimize effects of biological and analytical variability between the different lots, a normalization of retention time, signal intensity and mass was performed. In total, 292 signals for mass and CE-time with a frequency ≥35% could be determined that served as reference signals for normalization of peptide CE-time using local regression. The detected signal intensities for each individual peptide were normalized to the total ion count (total intensity) per individual sample analysis (ppm normalization).

All normalized peptides were deposited, matched, and annotated in a Microsoft SQL database, allowing further analysis and comparison of multiple samples [17]. Peptides were considered identical when deviation of mass was <±50 ppm (parts per million) for an 800 Da protein fragment, respectively <±75 ppm for a 15 kDa protein fragment. Peptides were considered identical if the CE-migration time window did not exceed 2–5%, continuously increasing between 10 and 60 min.

### Peptide sequencing

Nine lyophilized, tryptic-digested randomly selected vitreous samples were dissolved in 15 μL distilled water for MS/MS analysis. Separation was carried out via Dionex Ultimate 3000 RSLS nano flow system (Dionex, Camberly UK) as described by Metzger *et al*. [18]. After loading 5 μl onto a Dionex 0.1×20 mm 5 μm C18 nano trap column at a flowrate of 5 μl/min in 98% 0.1% formic acid and 2% acetonitrile, sample was eluted onto an Acclaim PepMap C18 nano column 75 μm×15 cm, 2 μm 100 Å at a flow rate of 0.3 μl/min. The trap and nano flow column were maintained at 35°C. The samples were eluted with a gradient of solvent A: 98% 0.1% formic acid, 2% acetonitrile versus solvent B: 80% acetonitrile, 20% 0.1% formic acid starting at 1% B for 5 minutes rising to 20% B after 90 min and finally to 40% B after 120 min. Subsequently, the column was washed and re-equilibrated prior to the next injection. The Proxeon nano spray ESI source (Thermo Fisher Hemel UK) in positive ion mode was used for samples ionisation. Ionization voltage was 2.6 kV, the capillary temperature was 200°C. The samples were analysed using an LTQ Orbitrap Velos (Thermo Finnigan, Bremen, Germany). The mass spectrometer was operated in MS/MS mode scanning from 380 to 2000 amu. The samples were analysed using both CID and HCD to obtain the maximum number of sequence identifications. The top 20 multiply charged ions were selected from each scan for MS/MS analysis using either CID or HCD at 40% collision energy. The resolution of ions in MS1 was 60,000 and 7,500 for mass fragmentation MS2. The searches were performed by the use of Proteome Discoverer (version 1.3, Thermo Fisher Scientific, Bremen, Germany) with the use of the SEQUEST algorithm and against the human, non redundant IPI database (version 3.87, entry count: 91464) for each data file separately. Trypsin was used as the enzyme while screening for proteins. Hydroxylated proline from collagen fragments and oxidation of methionine were accepted as variable modifications and carbamidomethylated cysteine as fixed modification. A maximal mass deviation of 10 ppm for precursor ions and 0.8 Da for product ions was accepted. The allowed false discovery rate was 1% and the number missed cuts one. In addition, only peptides with medium or high confidence, rank one and XCorr factor >0.8 were accepted.

The sequences were matched to the detected CE-MS data according to Zürbig *et al*. [19]. Charge of the peptides was used in this matching procedure instead of the LC retention time. On the basis of their charge, discrimination between peptides with similar masses can be performed even when having identical or very close masses in the liquid chromatography coupled with tandem mass spectrometry (LC-MS/MS) analysis. This is due to the fact that the number of basic and neutral polar amino acids of peptide sequences distinctly correlates with their CE-MS migration time/molecular weight coordinates. This enables linking a unique LC-MS/MS peptide to a CE-MS peptide in nearly all cases [19]. This procedure has been successfully used in a number of studies linking specific CE-MS-identified peptides to sequences obtained by LC-MS/MS [12,18,20–22].

CE-MS peptides with sequencing information were combined for each protein. Proteins were accepted when being represented by a minimum of two peptides. Protein abundance was calculated as the average of all normalized CE-MS peptide intensities for the given protein.

### ELISA

Clusterin, Complement C3, Ig lambda-like polypeptide 5 (IGLL5), Opticin and Vitronectin concentrations were assayed in sandwich-ELISAs (Cloud-Clone Corporation, Houston, TX, USA) where antibodies specific to the target proteins were precoated to a microtiterplate. After antigen binding to the precoated plates biotin-conjugated antibodies specific for Clusterin, Complement C3, Opticin or Vitronectin were used for detection of the bound antigens. Horseradish peroxidase conjugated to avidine and TMB substrate were applied for quantification. Measurements were conducted spectrophotometrically at 450nm. Minimum detectable doses were 3 ng/ml for Clusterin, 2 ng/ml for Complement C3, 0,2 ng/ml for IGLL5, 0,1 ng/ml for Opticin, and 0,5 pg/ml for Vitronectin.

### Statistical methods

SPSS version 21.0 was used for statistical analysis. A *P*-value of α<5.00E-02 was considered statistically significant.

#### Descriptive Statistics

For the descriptive data analysis, mean ± standard deviation (SD), median values, and minimal and maximal values were calculated.

#### Patient Characterization

Clinical and demographic characteristics of control patients and each subgroup of RVO (CRVO, H-CRVO, BRVO) was compared by using the Kruskal Wallis test where appropriate.

#### Proteomic Analysis

Candidate RVO biomarkers were defined by examination of differences in signal intensity of the proteins between the RVO-patients and the controls. Mean CE-MS based protein signal intensity was used as a measure for relative abundance. Mann-Whitney test was used for analysis. Multiple hypotheses testing correction was performed by using the Benjamini-Hochberg test for false discovery rate [23].

#### Subgroup Analysis

**Fig 1:**
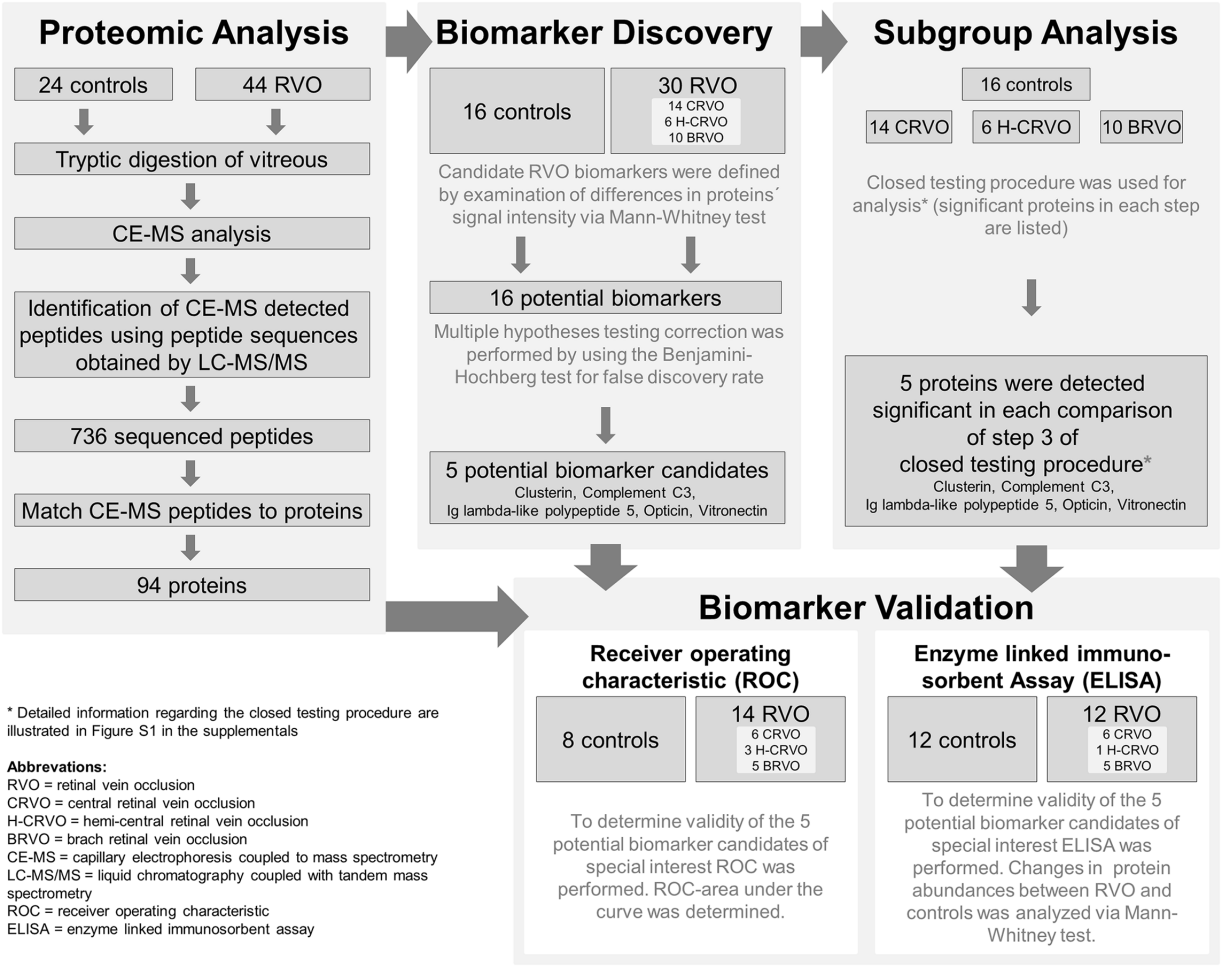
Study layout.

Patients with RVO were subdivided in following subgroups: CRVO, H-CRVO, BRVO. The closed testing procedure was used for subgroup analysis [24]. For detailed information see Fig 1.

#### Influence of past time period since suffering RVO on biomarker discovery

To analyze the influence of past time period since suffering RVO we compared protein signal intensity between fresh (patients suffered RVO <8 weeks ago) and old RVO-samples (patients suffered RVO ≥8 weeks ago) *versus* controls. The Mann-Whitney test was used for analysis.

#### Validation of Selected Potential Biomarker Candidates

To determine validity of potential biomarker candidates in RVO, receiver operating characteristic (ROC) was performed by using data of randomly selected, independent control and RVO samples not being used in proteomic analysis and biomarker definitions. Area under the curve (AUC) was determined. Furthermore, the protein abundance in VH of the selected potential biomarkers was measured by ELISA. Mann-Whitney test was used to analyze changes in the protein abundance between RVO and controls in the ELISA.

## Results

### Patient characterization

A total of 44 RVO samples and 24 controls with idiopathic floaters were analyzed. Patients with RVO were additionally subdivided in the following subgroups: CRVO (n = 20), H-CRVO (n = 9), BRVO (n = 15). Epidemiologic data and further patient information such as fresh/old RVO, lens status, retinal thickness, attachment of the vitreous body, and presence of retinal cysts are listed in Table 1 (for detailed information see S1 Table). No differences between subgroups regarding patient-age and average or center retinal thickness were observed (*P* = 1.60E-01 to 5.48E-01).

**Table 1:** Epidemiology.

	CRVO	Hemi-CRVO	BRVO	Control
**N**	20	9	15	24
**Fresh/Old**	14/6	7/2	11/4	-
**Female/Male**	8/12	4/5	10/5	13/11
**Age in years (mean ± SD)**	67.6 ± 14.3*	69.2 ± 14.2*	68.4 ± 12.2*	62.7 ± 11.7*
**Phakic/Pseudophakic**	16/4	8/1	13/2	12/12
**Average Thickness in μm (mean ± SD)**	376.3 ± 82.8*	351.33 ± 44.2*	351.5 ± 69.6*	-
**Center Thickness in μm (mean ± SD)**	513.8 ± 196.9*	443.4 ± 142.2*	462.0 ± 149.9*	-
**Posterior Vitreous attached/ detached/unrecognizable**	6/14/0	3/5/1	0/14/1	-
**Cysts yes/no**	15/5	8/1	13/2	-

* Kruskal Wallis test was used for analyses; *P*-Value of α<5.00E-02 was considered significant. No significant difference was tested: Age *P* = 3.71E-01; Average Thickness *P* = 1.60E-01; Center Thickness *P* = 5.48E-01

### Proteomic analysis

The study layout is depicted in Fig 1. Samples were randomly subdivided for selection and verification of potential biomarker candidates. Two thirds of all samples were used as a discovery set and selection of potential biomarker candidates and subgroup analysis (16 controls, 14 CRVO, 6 H-CRVO, 10 BRVO); one third was used for the validation using ROC analysis (8 controls, 6 CRVO, 3 H-CRVO, 5 BRVO). The two mass spectrometry techniques employed in this study are used in a complementary fashion. CE-MS, due to its high reproducibility, is used for candidate peptide selection while LC-MS/MS is used for identification of the biomarker peptides. Using LC-MS/MS analysis, 736 of the tryptic peptides detected with CE-MS could be sequenced. The mass of sequenced peptides ranged between 800.4 and 3619.0 Da. Migration time ranged between 20.1 and 38.0 min. The 736 tryptic peptides corresponded to 94 different proteins in VH (S2 Table, Fig 1). For detailed information regarding all raw data see S3 Table. In the interest of greater clarity, we deleted keratins (8 proteins, 72 peptides) and dermcidin (2 peptides) from the list since these proteins are only artifacts and aren’t proteins of vitreous humor. For all raw data see ftp://PASS00883:PZ4442mcv@ftp.peptideatlas.org.

### Selection of potential biomarker candidates

In the discovery study, the comparison of protein data between RVO (irrespective of subgroup) and control samples resulted in 16 significant proteins (*P* = 1.43E-05 to 4.48E-02; Table 2). After using correction for multiple testing, five proteins remained significant (Table 2, see *; Fig 1): Clusterin, Complement C3, IGLL5, Opticin and Vitronectin.

**Table 2:** Significant proteins in vitreous humor. Significant proteins were detected **by** capillary electrophoresis coupled to mass spectrometer (CE-MS) and identified by tandem mass spectrometry (LC-MS/MS) when comparing protein signal intensity of retinal vein occlusion (RVO)-samples compared to control-samples.

	Protein	UniProt^‡^	RVO (n = 30)					Control (n = 16)					*P*-Value^§^	Benjamini-Hochberg (adjusted *P*-Value)
			Protein identification		Statistical analysis			Protein identification		Statistical analysis				
			Pep-tides^##^	Cover-age^†^	N^#^	Mean intensity	SD	Pep-tides^##^	Cover-age^†^	N^#^	Mean intensity	SD		
**Upregulation**	Ig lambda-like polypeptide 5*	B9A064	3	20	30	358.2	422.5	2	13	11	93.4	75.7	1.43E-05	1.37E-03
	Vitronectin*	P04004	4	10	25	89.6	136.0	3	8	8	8.8	11.0	2.74E-04	8.77E-03
	Clusterin*	P10909	9	26	30	524.1	194.2	10	28	15	282.3	174.6	4.97E-04	1.19E-02
	Complement C3*	P01024	43	29	30	582.3	134.3	41	27	16	390.2	194.5	1.85E-03	3.55E-02
	Collagen alpha-2(XI) chain	P13942	3	6	30	740.4	678.7	5	9	13	377.1	528.5	8.55E-03	1.10E-01
	Collagen alpha-1(VII) chain	Q02388	3	2	5	29.3	101.6	2	1	8	25.1	57.2	2.94E-02	1.88E-01
	Collagen alpha-2(I) chain	P08123	4	7	18	15.1	15.8	4	7	6	5.1	8.1	4.32E-02	2.53E-01
	Collagen alpha-1(III) chain	P02461	3	4	23	93.4	91.6	3	4	10	37.9	41.8	4.48E-02	2.53E-01
**Downregulation**	Opticin*	Q9UBM4	6	20	23	82.8	207.2	5	16	16	163.7	95.1	7.07E-05	3.39E-03
	Neuroblast differentiation associated protein AHNAK	Q09666	2	1	6	1.4	3.9	4	1	9	14.0	24.8	3.34E-03	5.34E-02
	Alpha-crystallin B chain	P02511	2	11	3	1.9	7.4	5	30	7	30.1	75.5	9.20E-03	1.10E-01
	Complement factor B	P00751	2	3	13	18.1	60.4	4	6	11	44.9	61.4	1.23E-02	1.31E-01
	Obscurin	Q5VST9	2	<1	21	520.3	1358.1	2	<1	16	1767.1	3857.7	1.51E-02	1.31E-01
	Apolipoprotein A-II	P02652	2	29	10	8.1	14.4	4	41	10	50.1	67.6	1.78E-02	1.41E-01
	Complement C4-A	P0C0L4	2	2	4	2.0	6.9	4	3	7	29.3	78.3	1.91E-02	1.41E-01
	Serum albumin	P02768	40	61	30	2109.7	657.6	42	65	16	2910.3	1163.1	2.11E-02	1.45E-01

^‡^ Listed in the universal protein resource (UniProt), a central repository of protein data.

^##^ Number of peptides observed by CE-MS analysis and sequenced by LC-MS/MS for each protein.

^†^ Percentage (%) of peptide coverage of the protein sequence.

^#^ Number of samples with a signal intensity >0

^§^ *P*-Value was analyzed by using the Mann-Whitney test. A *P* of α<5.00E-02 was considered statistically significant.

* Proteins which remained significant after performing multiple hypotheses testing correction, analyzed by using the Benjamini-Hochberg test for false discovery rate. An adjusted *P*-Value of α<5.00E-02 was considered statistically significant.

### Subgroup analysis

Signal intensity of the proteins of each subgroup (CRVO, H-CRVO, BRVO) and the controls was compared by using the closed testing procedure (see S1 Fig). Proteins being detected significantly different in any of the analysis are listed in Table 3. In step 3 ten proteins were expressed significantly different in each, CRVO and BRVO samples versus controls, 9 proteins were expressed significantly different in H-CRVO versus controls. Five proteins remained significant in all comparisons of step 1 to step 3: Clusterin, Complement C3, IGLL5, Opticin and Vitronectin. For detailed information regarding subgroup analysis see S4 Table.

**Table 3:** Significant proteins in vitreous humor of subgroups of retinal vein occlusion-samples compared to control-samples. Significant proteins were detected by capillary electrophoresis coupled to mass spectrometer (CE-MS) and identified by tandem mass spectrometry (LC-MS/MS) when comparing protein signal intensity of subgroups of retinal vein occlusion (RVO)-samples (central RVO (CRVO), hemi-central RVO (H-CRVO), branch RVO (BRVO)) compared to control-samples.

Protein	UniPort^‡^	Control (n = 16)			CRVO (n = 14)			*P*-Value^§^ Control vs. CRVO	H-CRVO (n = 6)			*P*-Value^§^ Control vs. H-CRVO	BRVO (n = 10)			*P*-Value^§^ Control vs. BRVO
		N^#^	Signal intensity		N^#^	Signal intensity			N^#^	Signal intensity			N^#^	Signal intensity		
			Mean	SD		Mean	SD			Mean	SD			Mean	SD	
Clusterin*^;^ ^†^^;^ ^††^^;^ ^†††^^;^ ^††††^	P10909	15	282.3	174.6	14	487.5	187.6	1.12E-02	6	570.4	248.9	9.87E-03	10	547.6	179.5	3.75E-03
Collagen alpha-1(V) chain^†^^;^ ^†††^	P20908	13	64.7	142.9	8	14.3	24.0	1.09E-01	5	34.8	23.2	3.55E-01	9	64.7	39.9	2.85E-02
Collagen alpha-2(XI) chain^†^^;^ ^††^^;^ ^†††^	P13942	13	377.1	528.5	14	1097.0	856.5	9.92E-03	6	371.7	40.9	1.84E-01	10	462.3	222.2	7.29E-02
Complement C3*^;^ ^†^^;^ ^††^^;^ ^†††^^;^ ^††††^	P01024	16	390.2	194.5	14	569.1	167.6	1.99E-02	6	603.5	84.2	1.83E-02	10	588.1	113.9	1.32E-02
Complement C4-A^†^^;^ ^††^^;^ ^†††^	P0C0L4	7	29.3	78.3	0	0.0	0.0	6.01E-03	2	7.5	14.6	6.79E-01	2	1.5	3.3	1.72E-01
Complement factor B^†^^;^ ^††^^;^ ^††††^	P00751	11	44.9	61.4	8	13.3	21.2	9.64E-02	1	1.2	3.0	1.82E-02	4	35.1	102.7	7.05E-02
Fibrinogen alpha chain^†^^;^ ^††^^;^ ^††††^	P02671	6	12.0	31.1	6	6.3	8.7	7.78E-01	6	18.7	9.9	2.02E-02	4	6.8	12.7	1.00E+00
Haptoglobin^†^^;^ ^†††^^;^ ^††††^	P00738	10	30.6	51.3	9	14.9	23.8	7.33E-01	5	24.3	21.1	5.00E-01	10	113.0	140.1	1.47E-02
Ig lambda-2 chain C regions^†^^;^ ^†††^^;^ ^††††^	P0CG05	12	452.7	305.4	10	484.6	494.0	9.67E-01	5	684.3	465.2	2.36E-01	10	1063.4	446.5	1.84E-03
IgGFc-binding protein^†^^;^ ^††^^;^ ^††††^	Q9Y6R7	14	181.5	268.0	9	170.1	443.2	1.22E-01	2	11.3	17.7	2.10E-02	9	106.6	115.0	6.73E-01
Ig lambda-like polypeptide 5*^;^ ^†^^;^ ^††^^;^ ^†††^^;^ ^††††^	B9A064	11	93.4	75.7	14	215.4	118.0	2.05E-03	6	561.9	619.6	1.11E-03	10	435.9	525.0	2.14E-04
Neuroblast differentiation-associated protein AHNAK^†^^;^ ^†††^^;^ ^††††^	Q09666	9	14.0	24.8	3	2.1	5.1	2.91E-02	3	2.4	3.5	2.62E-01	0	0.0	0.0	5.22E-03
Obscurin^†^^;^ ^††^^;^ ^††††^	Q5VST9	16	1767.1	3857.7	8	775.6	1967.0	3.00E-02	4	136.1	161.8	1.50E-02	9	393.5	349.5	2.92E-01
Opticin*^;^ ^†^^;^ ^††^^;^ ^†††^^;^ ^††††^	Q9UBM4	16	163.7	95.1	9	111.6	294.7	7.39E-04	6	62.6	61.7	1.83E-02	8	54.6	91.0	1.87E-03
Pigment epithelium-derived factor^†^^;^ ^†††^^;^ ^††††^	P36955	15	161.2	134.1	14	145.8	56.7	9.01E-01	6	177.5	55.4	3.38E-01	10	246.5	74.1	1.77E-02
Serum albumin^†^^;^ ^††^^;^ ^†††^	P02768	16	2910.3	1163.1	14	1759.2	603.5	6.08E-03	6	2385.6	325.2	2.69E-01	10	2434.6	670.3	3.17E-01
Vitronectin*^;^ ^†^^;^ ^††^^;^ ^†††^^;^ ^††††^	P04004	8	8.8	11.0	10	71.9	100.9	1.81E-02	6	38.3	16.0	1.51E-03	9	145.1	197.6	1.50E-03

^‡^ Listed in the universal protein resource (UniProt), a central repository of protein data.

^#^ Number of samples with a signal intensity >0

^§^ *P*-Value was analyzed by using the Mann-Whitney test. A *P* of α<5.00E-02 was considered statistically significant.

Closed testing procedure was used to verify the results. Kruskal-Wallis Test was used for analysis. A *P* of α<5.00E-02 was considered statistically significant.

Proteins are marked when being significant in listed steps: Step 1:

^†^ Control vs. CRVO vs. H-CRVO vs. BRVO

Step 2:

^††^ Control vs. CRVO vs. H-CRVO

^†††^ Control vs. CRVO vs. BRVO

^††††^ Control vs. H-CRVO vs. BRVO

Step 3: For detailed information of step 3 see §

* Proteins which remained significant after performing multiple hypotheses testing correction, analyzed by using the Benjamini-Hochberg test for false discovery rate.

### Influence of past time period since suffering RVO on biomarker discovery

When analyzing the influence of past time period since suffering RVO Clusterin, Ig lambda-like polypeptide 5 (IGLL5), Opticin and Vitronectin remained significantly different between control samples (n = 16) *versus* fresh (n = 22) or old RVO-samples (n = 8) (S5 Table). Except for Apolipoprotein A-II (*P* = 1.75E-02) no difference was detected of the potential biomarkers when comparing signal intensity of the proteins between the fresh *versus* old RVO-samples (*P* = 8.27E-02 to 9.68E-01; S5 Table).

### Verification of selected potential biomarker candidates

**Fig 2:**
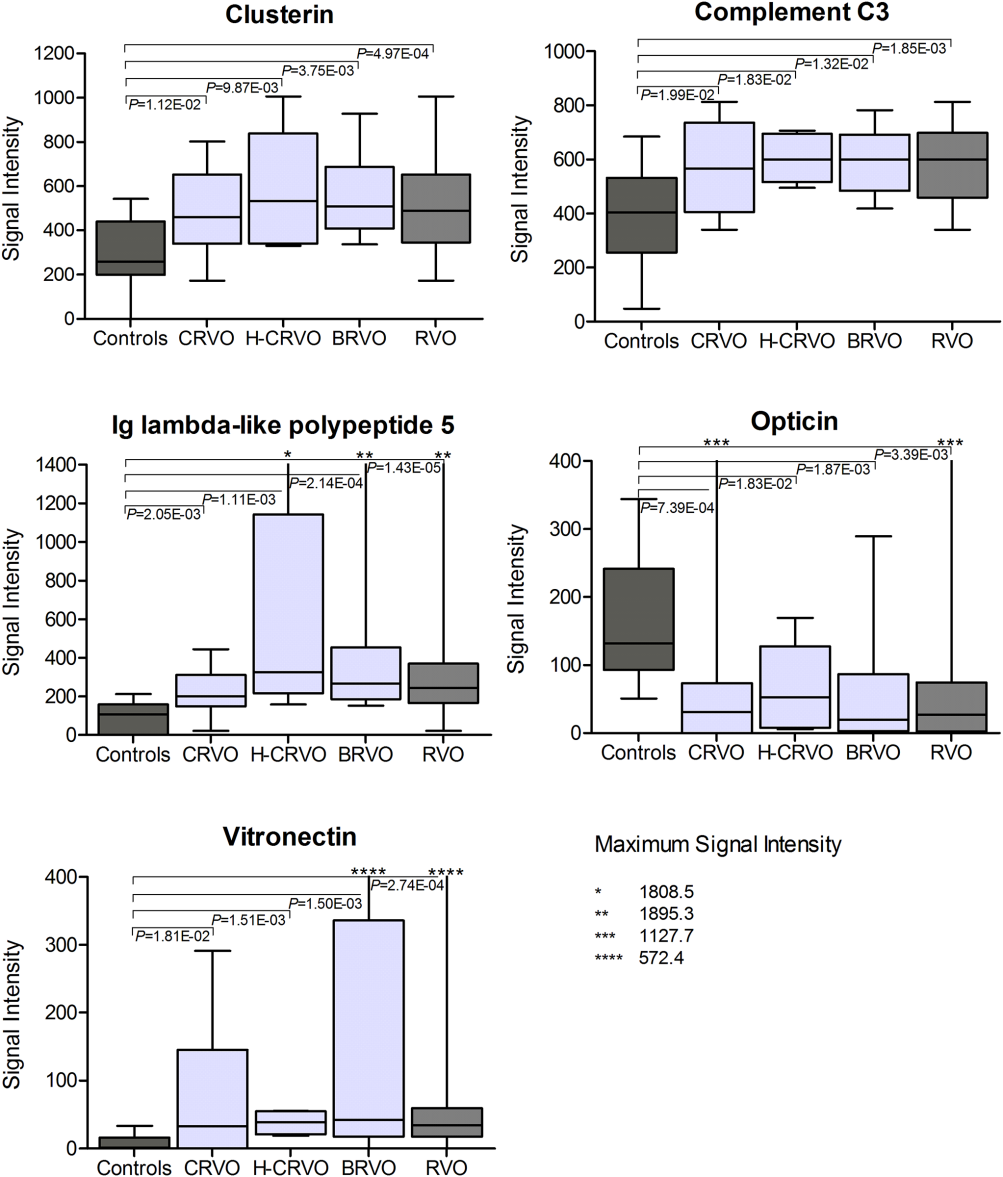
Comparison of signal intensity of potential biomarker proteins. Comparison of signal intensity of proteins remained significant after using correction for multiple testing (Benjamini-Hochberg test) when comparing retinal vein occlusion (RVO) versus controls (see *; Table 2) of samples used for biomarker candidates identification (discovery set). Mann-Whitney test was used for analysis. A *P* of α<5.00E-02 was considered statistically significant. RVO was subdivided in following subgroups: Central-RVO (CRVO), hemi-central RVO (H-CRVO), branch RVO (BRVO).

Fig 2 shows the comparison of signal intensity of Clusterin, Complement C3, IGLL5, Opticin and Vitronectin in the control group (n = 16) compared to the three subgroups in the discovery cohort (14 CRVO, 6 H-CRVO, 10 BRVO) and compared to the total discovery cohort (30 RVO). All these proteins remained significant after using correction for multiple testing when comparing RVO versus controls (Table 2) and were listed significant in each comparison of the subgroup analysis (Table 3). Therefore, these proteins were considered for verification of potential biomarker candidates (Fig 1).

**Fig 3:**
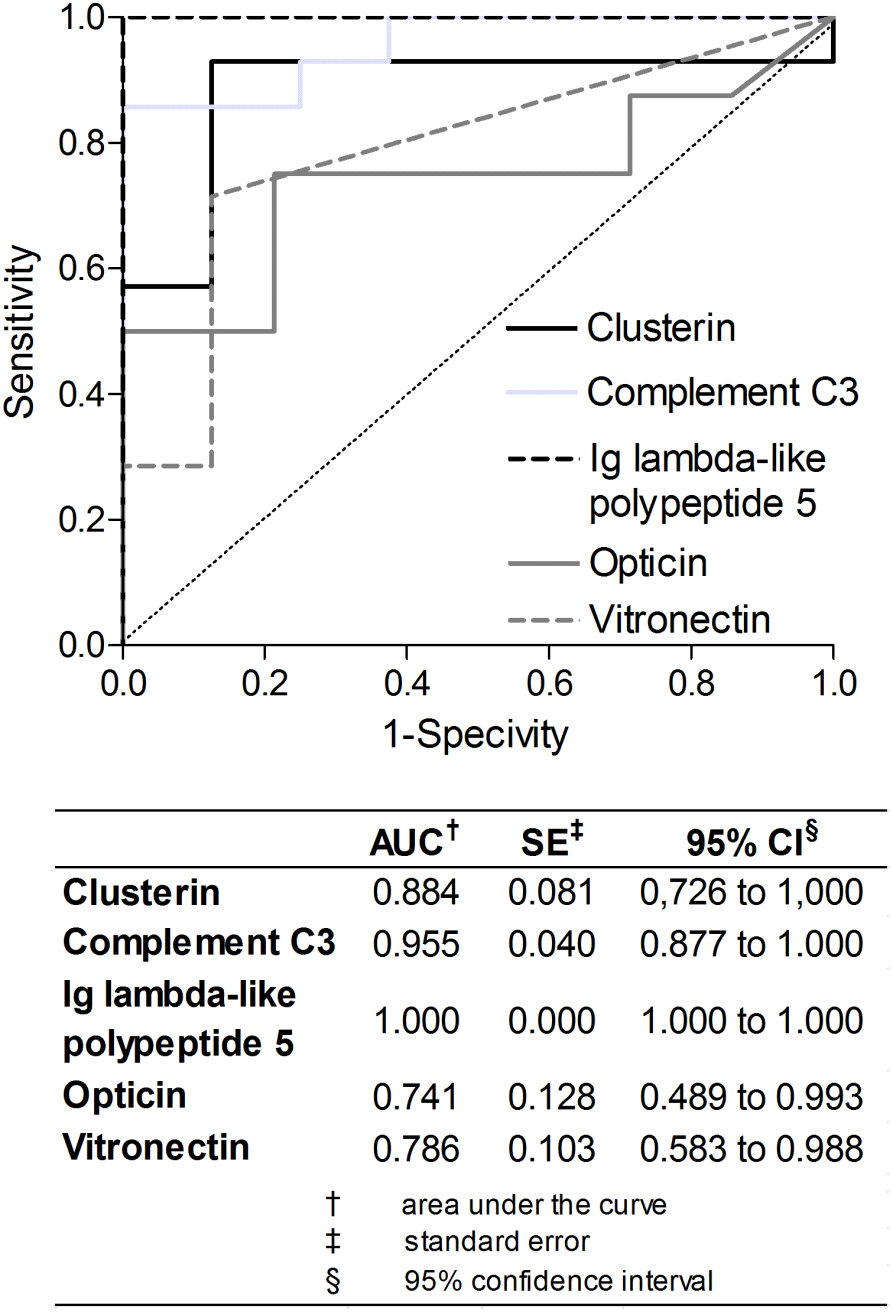
Biomarker validation—Receiver operating characteristic (ROC) curves of selected candidate proteins. Independent samples of 14 patients suffered a retinal vein occlusion (RVO) and 8 controls were used. The area under the curve (AUC) was shown for RVO at 95% confidence level (95% CI).

**Fig 4:**
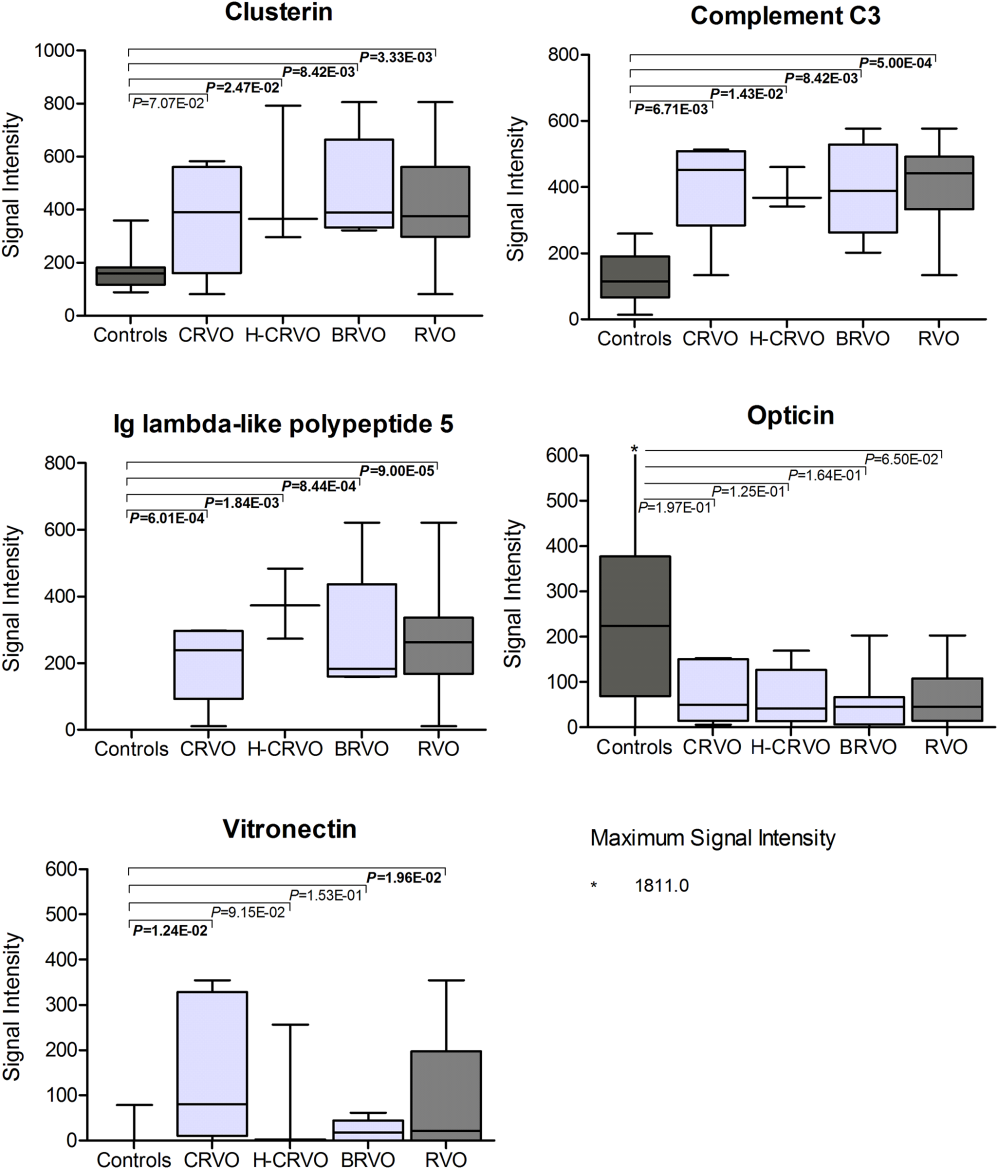
Comparison of signal intensity of proteins used for biomarker validation. Mann-Whitney test was used for analysis. A *P* of α<5.00E-02 was considered statistically significant. Significant values are written in bold. Retinal vein occlusion (RVO) was subdivided in following subgroups: Central-RVO (CRVO), hemi-central RVO (H-CRVO), branch RVO (BRVO).

To determine validity of potential biomarker candidates in RVO, we performed receiver operating characteristic (ROC) analysis (Fig 3) using randomly selected, independent samples. For analyses we used data of 8 controls and 14 RVO patients that were not included in analyses of biomarker definitions. AUCs were: Clusterin 0.884 (*P* = 3.34E-03), Complement C3 0.955 (*P* = 5.00E-04), IGLL5 1.000 (*P* = 1.32E-04), Opticin 0.741 (*P* = 6.54E-02), Vitronectin 0.786 (*P* = 2.90E-02). Fig 4 shows the comparison of signal intensity of the 5 proteins in the control group (n = 8) compared to the three subgroups in the discovery cohort (6 CRVO, 3 H-CRVO, 5 BRVO) and compared to the total discovery cohort (14 RVO).

The vitreal protein concentration of Clusterin, Complement C3, IGLL5, Opticin and Vitronectin was also measured using ELISA. Twelve RVO and twelve control samples were used. All Clusterin, Complement C3, Opticin and Vitronectin analyzed proteins were successfully detected in all vitreous samples. Unfortunately, we were not able to detect IGLL5 in any of the vitreous samples. Opticin displayed reduced abundance, whereas Clusterin, Complement C3 and Vitronectin showed increased abundance in the VH in the RVO group (see Table 4). Thus, the variations in the protein concentration observed by ELISA corresponded to the changes in signal intensity identified by mass spectrometry analysis. All four detected proteins showed a significant difference in the protein abundance between the RVO and the control group (see Table 4). Thus, the ELISA results further affirm our primary findings by mass spectrometry.

**Table 4:** Biomarker validation—Enzyme Linked Immunosorbent Assay (ELISA). Vitreal levels of our five potential biomarkers in retinal vein occlusion (RVO, n = 12) and control samples (n = 12) messured by ELISA.

Protein	UniProt^‡^	RVO (n = 12)					Control (n = 12)					P-Value^§^ RVO vs. Control
		N^#^	Mean	SD	Min	Max	N^#^	Mean	SD	Min	Max	
Clusterin (ng/ml)	P10909	12	16.7	2.4	12.7	19.9	12	6.6	2.0	3.2	9.9	3.12E-05
Complement C3 (ng/ml)	P01024	12	3659.6	336.0	3188.0	4111.0	12	3241.3	464.3	2268.4	3657.8	3.77E-02
Immunoglobulin lambda-like polypeptide	B9A064	0	-	-	-	-	0	-	-	-	-	-
Opticin (pg/ml)	Q9UBM	12	524.3	83.6	412.0	632.0	12	579.7	90.2	450	734	4.94E-02
Vitronectin (ng/ml)	P04004	12	74.3	21.7	51.8	117.0	12	26.2	17.7	8.1	55.7	1.36E-04

^‡^ Listed in the universal protein resource (UniProt), a central repository of protein data.

^#^ Number of samples in which the proteins were detected with ELISA

^§^ *P*-Value was analyzed by using the Mann-Whitney test. A *P* of α<5.00E-02 was considered statistically significant

## Discussion

The aim of this study was to analyze the protein profile of undiluted human vitreous of untreated patients with RVO and to identify potential biomarkers that are associated with the pathophysiology of the disease. CE-MS and LC-MS/MS were used for sample analysis in this study. CE-MS is a powerful and very reproducible technology platform with known performance characteristics [25] and therefore is used as one of the most advanced techniques for the discovery of new protein biomarkers of clinical significance [25–28]. Furthermore, CE-MS allows the characterization of highly complex samples in a consistent and reproducible way and has a high reproducibility allowing the comparison of the protein content of samples over time [25,29]. LC-MS/MS was used in this study to provide sequence information (i.e. identify) of the CE-MS detected peptides.

### Potential biomarker candidates

Comparing the proteome of 30 RVO patients and 16 controls, we could define 16 proteins that were significantly up- or downregulated in RVO. Thrombosis and thrombolysis are involved in RVO as well as inflammatory processes due to hypoxic induced cell death [30]. This could be confirmed in our study as inflammatory response pathway, complement activation (classical pathway) as well as complement and coagulation cascades were identified as primary pathways of the 16 significant up- or downregulated proteins in VH of RVO patients compared to controls.

We believe, that five of the 16 significant proteins, Clusterin, Complement C3, IGLL5, Opticin and Vitronectin, are of special interest as they remained significant after multiple testing, analysis of fresh *versus* old RVO, and subgroup analysis (CRVO, H-CRVO, BRVO *versus* controls).

Clusterin is a glycoprotein with a nearly ubiquitous tissue distribution and an apparent involvement in a number of biological processes, including *inter alia* lipid transport, membrane recycling, cell adhesion, programmed cell death, and complement cascade [31,32]. Interestingly, increased blood levels of Clusterin are associated with atrophy of the entorhinal cortex in Alzheimer’s disease for which this protein seems to be a marker of disease severity [33]. Therefore, upregulated Clusterin levels may indicate biochemical signs of a neurodegenerative disease. Our findings indicate for the first time that Clusterin in VH is associated with retinal disease like RVO.

Complement C3 plays a central role in the activation of complement system. Among other things, it is involved in the adaptive immune response to select the appropriate antigens for a humoral response, promotes phagocytosis and supports local inflammatory responses against pathogens [34]. Complement C3 might thus be involved in the well known phagocytic and inflammatory process subsequent to the onset of RVO.

Similar to Complement C3, IGLL5 demonstrated upregulation in RVO. IGLL5 could also be involved in immune processes being associated with cell death due to RVO although IGLL5 seems not to be expressed in pre-B-cells (http://uniprot.org). In general little is known about IGLL5 in the literature and further research is needed to explain the association of high IGLL5 levels in VH of patients with RVO.

Opticin is a protein produced by the non-pigmented ciliary epithelium. It is present in significant quantities in the vitreous of the eye and also localizes at the cornea, the iris, the ciliary body, the optic nerve, the choroid and the retina. It can noncovalently bind collagen fibrils and regulate fibril morphology, spacing, and organization [35]. Furthermore, Opticin was found to be located, besides various ocular tissues, especially in basal and cortical vitreous and adjacent basement membranes, like the internal limiting membrane, suggesting a possible role in vitreoretinal adhesion [36]. Therefore, decreased Opticin levels may be correlated with macula edema due to RVO.

Vitronectin is an abundant glycoprotein which promotes cell adhesion and spreading, inhibits the membrane damaging effect of the terminal cytolytic complement pathway, and binds to several serpins. By its localization in the extracellular matrix and its binding to plasminogen activation inhibitor-1, vitronectin can potentially regulate the proteolytic degradation of this matrix. In addition, vitronectin binds to complement, to heparin and to thrombin-antithrombin III complexes, showing its participation in the immune response and in the regulation of clot formation [37]. Therefore, upregulation of Vitronectin in RVO potentially plays a role in the disease.

### Advantages and limitations of the study

To our knowledge, this is the first proteomics study using a high number (n = 30 and 16 idiopathic vitreous floaters as controls) of undiluted VH of RVO patients and validation of the 5 most significant proteins in an independent data set of an additional 8 controls and 14 RVO patients. We could only confirm 3 of the proteins 36 different proteins of the Yao *et al*. study [14] (Alpha-crystallin B chain, Beta-crystallin B2, Serum albumin) mostly due to this difference in power and the fact that they used 2-dimensional electrophoresis coupled with MS providing a different coverage of the proteome. Furthermore, we compared our results with other high throughput studies on VH. We have identified 85 proteins by other studies of vitreous humor proteome [12,38–40]. In addition, we have identified 9 novel VH proteins (see S2 Table) not described in previous studies, although our study fails to detect more than 94 proteins compared to the studies of Aretz et al. and Murthy et al. which detected in total 1111, respectively 1205 proteins in VH.

Nevertheless, there are some limitations of our study. First of all, there might be some bias in biomarker definitions by using patients with idiopathic vitreous floaters as controls. The existence of idiopathic vitreous floaters might have an influence on protein profile in vitreous body and therefore might have an influence of biomarker definitions in this study. Furthermore, highly abundant VH proteins such as albumin and immunoglobulin were not depleted in this study, possibly preventing the detection of less abundant proteins [41]. This seems to be the reason why only 94 proteins were identified although one would expect a higher number of proteins in human VH and why the number of detected peptides and coverage is low for some proteins (see S2 Table and Table 2), limiting statistical analysis of these proteins. In addition, depletion of highly abundant proteins does not necessarily lead to increased detection of low abundance proteins. On the contrary it has been shown that depletion can lead to co-depletion of many other proteins as shown for the analysis of the plasma proteome [42]. Another limitation of our study is the high variability of the mean intensity in some identified proteins resulting in a high SD (see for example Tables 2 and 3). The high variability may be induced by accepting identification of proteins when being represented by only two peptides. In future studies variability of the mean intensity of the proteins could be reduced by accepting identification of proteins when being represented in more than two peptides or by higher group sizes, leading to the next limitation of our study, the limited subgroup sizes. Although performing proteomic analysis of in total 68 participants (Proteomic analysis: 30 RVO, 16 controls; ROC analysis: 14 RVO, 8 controls) bigger group sizes would be desirable to further improve the power. Furthermore, with bigger group sizes one would expect significant results in all analyses of verification of potential biomarker candidates.

## Conclusions

In this retrospective, clinical-experimental study we applied a reproducible proteomics detection method in a high number of undiluted vitreous humor of patients with RVO. Our findings were thoroughly statistically evaluated and yielded five potential biomarker candidates associated with the pathophysiology of RVO: Clusterin, Complement C3, IGLL5, Opticin and Vitronectin. Future studies are needed to validate our substantial findings, which might be helpful regarding future diagnostic or therapeutic approaches in RVO. These studies should deplete highly abundant VH proteins such as albumin and immunoglobulin to determine more precisely potential biomarker candidates associated with the pathophysiology of RVO.

## Supporting Information

S1 Fig Closed testing procedure. (TIF)

S1 Table Epidemiology—detailed patient information. (DOCX)

S2 Table 94 proteins being detected in vitreous humor. (DOCX)

S3 Table Raw data. (XLS)

S4 Table Subgroup analysis—detailed information. (DOCX)

S5 Table Influence of past time period since suffering retinal vein occlusion on biomarker discovery. (DOCX)
