# Supplementary figures and images for: Proteomic Analysis of Vitreous Humor in Retinal Vein Occlusion
Source: PLoS One. 2016 Jun 30;11(6):e0158001. doi: 10.1371/journal.pone.0158001 (PMC4928959; doi:10.1371/journal.pone.0158001)

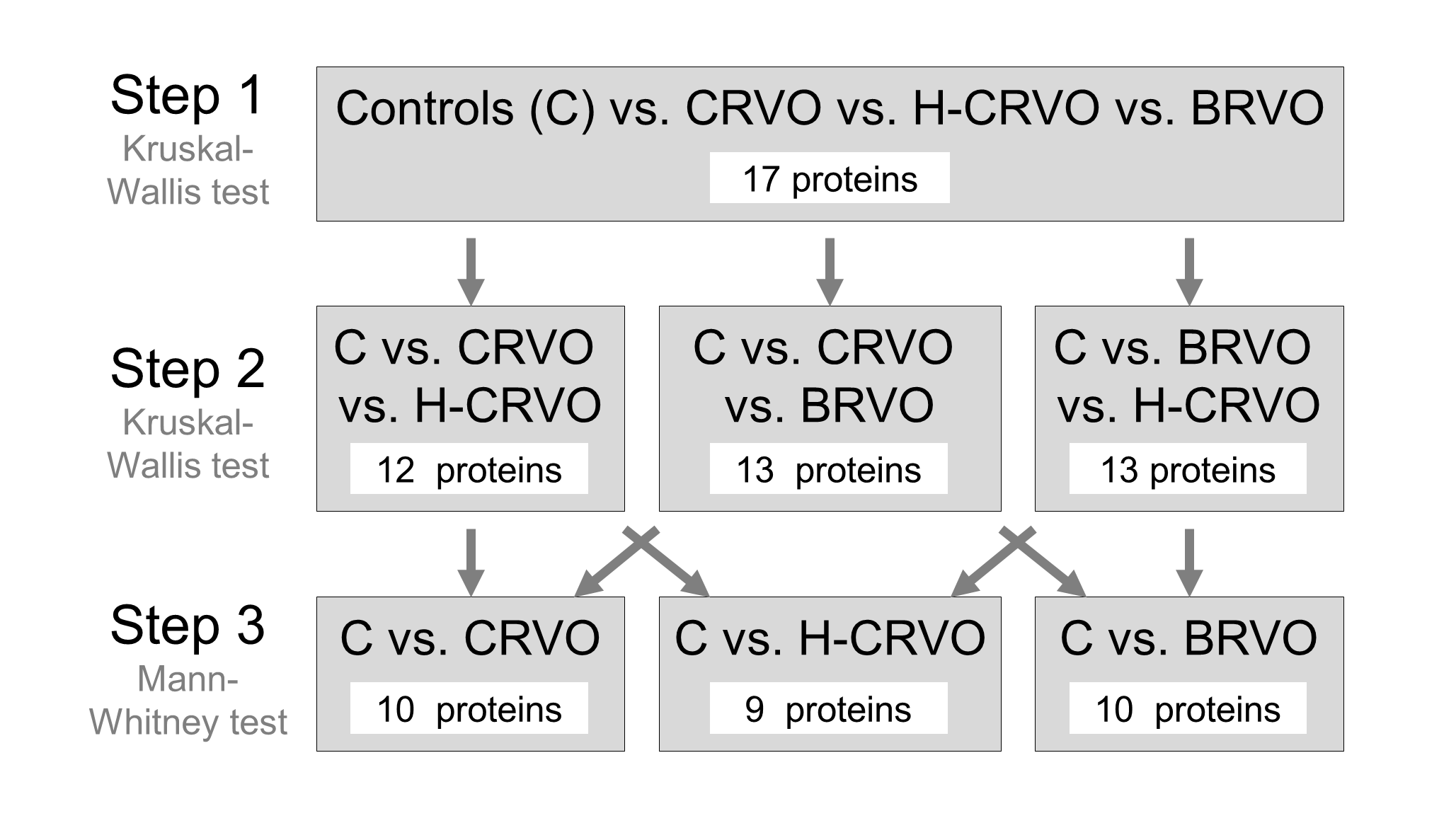

Supplement: S1 Fig — (TIF) [file pone.0158001.s001.tif]
