# Supplementary material for: Proteomic Analysis of Vitreous Humor in Retinal Vein Occlusion
Source: PLoS One. 2016 Jun 30;11(6):e0158001. doi: 10.1371/journal.pone.0158001 (PMC4928959; doi:10.1371/journal.pone.0158001)
Supplement: S1 Table — (DOCX) [file pone.0158001.s002.docx]

Table S1. Epidemiology - detailed patient information

|  |  |  | **Fresh/Old** | **Femal/Male** | **Age (years)** | **Phakic/Pseudophakic** | **Average Thickness (μm)** | **Center Thickness (μm)** | **Posterior Vitreous**  **attached/ detached/unrecognizable** | **Zysts yes/no** |
| --- | --- | --- | --- | --- | --- | --- | --- | --- | --- | --- |
| **Proteomic Analysis** | | | | | | | | | | |
|  | CRVO | |  |  |  |  |  |  |  |  |
|  |  | Sample 1 | 1/0 | 0/1 | 80 | 1/0 | 286 | 267 | 0/1/0 | 1/0 |
|  |  | Sample 2 | 0/1 | 1/0 | 84 | 0/1 | 322 | 384 | 0/1/0 | 1/0 |
|  |  | Sample 3 | 0/1 | 0/1 | 76 | 0/1 | 332 | 356 | 1/0/0 | 0/1 |
|  |  | Sample 4 | 0/1 | 0/1 | 54 | 1/0 | 430 | 430 | 0/1/0 | 1/0 |
|  |  | Sample 5 | 1/0 | 1/0 | 85 | 1/0 | 386 | 406 | 0/1/0 | 1/0 |
|  |  | Sample 6 | 1/0 | 1/0 | 83 | 0/1 | 103 | 621 | 1/0/0 | 1/0 |
|  |  | Sample 7 | 1/0 | 0/1 | 84 | 0/1 | 487 | 663 | 0/1/0 | 1/0 |
|  |  | Sample 8 | 1/0 | 0/1 | 53 | 1/0 | 374 | 420 | 0/1/0 | 0/1 |
|  |  | Sample 9 | 1/0 | 0/1 | 52 | 1/0 | 430 | 759 | 0/1/0 | 1/0 |
|  |  | Sample 10 | 1/0 | 0/1 | 55 | 1/0 | 405 | 697 | 0/1/0 | 0/1 |
|  |  | Sample 11 | 0/1 | 0/1 | 53 | 1/0 | 387 | 672 | 1/0/0 | 1/0 |
|  |  | Sample 12 | 1/0 | 1/0 | 81 | 1/0 | 371 | 985 | 0/1/0 | 0/1 |
|  |  | Sample 13 | 1/0 | 0/1 | 76 | 1/0 | 462 | 274 | 0/1/0 | 1/0 |
|  |  | Sample 14 | 1/0 | 1/0 | 60 | 1/0 | 417 | 708 | 0/1/0 | 0/1 |
|  |  | |  |  |  |  |  |  |  |  |
|  | Hemi-CRVO | |  |  |  |  |  |  |  |  |
|  |  | Sample 1 | 0/1 | 1/0 | 86 | 1/0 | 354 | 330 | 0/1/0 | 1/0 |
|  |  | Sample 2 | 1/0 | 1/0 | 72 | 1/0 | 377 | 527 | 1/0/0 | 1/0 |
|  |  | Sample 3 | 1/0 | 1/0 | 81 | 0/1 | 242 | 264 | 0/1/0 | 1/0 |
|  |  | Sample 4 | 1/0 | 0/1 | 64 | 1/0 | 375 | 376 | 1/0/0 | 1/0 |
|  |  | Sample 5 | 0/1 | 0/1 | 86 | 1/0 | 360 | 365 | 0/0/1 | 1/0 |
|  |  | Sample 6 | 1/0 | 0/1 | 63 | 1/0 | 345 | 551 | 0/1/0 | 0/1 |
|  |  |  |  |  |  |  |  |  |  |  |
|  | BRVO | |  |  |  |  |  |  |  |  |
|  |  | Sample 1 | 1/0 | 1/0 | 67 | 1/0 | 362 | 607 | 0/1/0 | 1/0 |
|  |  | Sample 2 | 1/0 | 1/0 | 68 | 1/0 | 440 | 599 | 0/1/0 | 1/0 |
|  |  | Sample 3 | 1/0 | 1/0 | 81 | 1/0 | 491 | 631 | 0/1/0 | 1/0 |
|  |  | Sample 4 | 1/0 | 1/0 | 81 | 1/0 | 301 | 482 | 0/0/1 | 1/0 |
|  |  | Sample 5 | 1/0 | 0/1 | 67 | 1/0 | 367 | 701 | 0/1/0 | 1/0 |
|  |  | Sample 6 | 0/1 | 1/0 | 83 | 0/1 | 279 | 273 | 0/1/0 | 1/0 |
|  |  | Sample 7 | 1/0 | 0/1 | 72 | 0/1 | 317 | 534 | 0/1/0 | 1/0 |
|  |  | Sample 8 | 1/0 | 0/1 | 77 | 1/0 | 303 | 316 | 0/1/0 | 0/1 |
|  |  | Sample 9 | 1/0 | 1/0 | 42 | 1/0 | 304 | 563 | 0/1/0 | 1/0 |
|  |  | Sample 10 | 0/1 | 0/1 | 52 | 1/0 | 369 | 394 | 0/1/0 | 1/0 |
|  |  |  |  |  |  |  |  |  |  |  |
|  | Controls | |  |  |  |  |  |  |  |  |
|  |  | Sample 1 | - | 1/0 | 78 | 0/1 | - | - | - | - |
|  |  | Sample 2 | - | 0/1 | 73 | 1/0 | - | - | - | - |
|  |  | Sample 3 | - | 0/1 | 51 | 0/1 | - | - | - | - |
|  |  | Sample 4 | - | 0/1 | 80 | 0/1 | - | - | - | - |
|  |  | Sample 5 | - | 1/0 | 54 | 1/0 | - | - | - | - |
|  |  | Sample 6 | - | 1/0 | 54 | 0/1 | - | - | - | - |
|  |  | Sample 7 | - | 1/0 | 68 | 1/0 | - | - | - | - |
|  |  | Sample 8 | - | 0/1 | 64 | 1/0 | - | - | - | - |
|  |  | Sample 9 | - | 1/0 | 74 | 0/1 | - | - | - | - |
|  |  | Sample 10 | - | 1/0 | 81 | 1/0 | - | - | - | - |
|  |  | Sample 11 | - | 1/0 | 52 | 0/1 | - | - | - | - |
|  |  | Sample 12 | - | 0/1 | 65 | 1/0 | - | - | - | - |
|  |  | Sample 13 | - | 1/0 | 65 | 1/0 | - | - | - | - |
|  |  | Sample 14 | - | 1/0 | 55 | 0/1 | - | - | - | - |
|  |  | Sample 15 | - | 0/1 | 67 | 0/1 | - | - | - | - |
|  |  | Sample 16 | - | 0/1 | 54 | 0/1 | - | - | - | - |
|  |  |  |  |  |  |  |  |  |  |  |
| **Biomarker Validation** | | | | | | | | | | |
|  | CRVO | |  |  |  |  |  |  |  |  |
|  |  | Sample 15 | 1/0 | 1/0 | 78 | 1/0 | 392 | 451 | 0/1/0 | 1/0 |
|  |  | Sample 16 | 1/0 | 1/0 | 76 | 1/0 | 406 | 511 | 1/0/0 | 1/0 |
|  |  | Sample 17 | 1/0 | 0/1 | 40 | 1/0 | 433 | 424 | 0/1/0 | 1/0 |
|  |  | Sample 18 | 1/0 | 0/1 | 59 | 1/0 | 366 | 353 | 1/0/0 | 1/0 |
|  |  | Sample 19 | 0/1 | 1/0 | 70 | 1/0 | 439 | 659 | 0/1/0 | 1/0 |
|  |  | Sample 20 | 0/1 | 0/1 | 52 | 1/0 | 300 | 235 | 1/0/0 | 1/0 |
|  |  |  |  |  |  |  |  |  |  |  |
|  | Hemi-CRVO | |  |  |  |  |  |  |  |  |
|  |  | Sample 7 | 1/0 | 0/1 | 73 | 1/0 | 356 | 689 | 1/0/0 | 1/0 |
|  |  | Sample 8 | 1/0 | 0/1 | 51 | 1/0 | 355 | 328 | 0/1/0 | 1/0 |
|  |  | Sample 9 | 1/0 | 1/0 | 47 | 1/0 | 398 | 561 | 0/1/0 | 1/0 |
|  |  |  |  |  |  |  |  |  |  |  |
|  | BRVO | |  |  |  |  |  |  |  |  |
|  |  | Sample 11 | 0/1 | 1/0 | 69 | 1/0 | 233 | 275 | 0/1/0 | 1/0 |
|  |  | Sample 12 | 1/0 | 1/0 | 80 | 1/0 | 305 | 540 | 0/1/0 | 1/0 |
|  |  | Sample 13 | 1/0 | 1/0 | 74 | 1/0 | 365 | 378 | 0/1/0 | 1/0 |
|  |  | Sample 14 | 1/0 | 0/1 | 61 | 1/0 | 394 | 213 | 0/1/0 | 0/1 |
|  |  | Sample 15 | 0/1 | 1/0 | 52 | 1/0 | 441 | 424 | 0/1/0 | 1/0 |
|  |  |  |  |  |  |  |  |  |  |  |
|  | Controls | |  |  |  |  |  |  |  |  |
|  |  | Sample 17 | - | 1/0 | 43 | 1/0 | - | - | - | - |
|  |  | Sample 18 | - | 0/1 | 62 | 0/1 | - | - | - | - |
|  |  | Sample 19 | - | 0/1 | 41 | 1/0 | - | - | - | - |
|  |  | Sample 20 | - | 1/0 | 52 | 1/0 | - | - | - | - |
|  |  | Sample 21 | - | 1/0 | 72 | 1/0 | - | - | - | - |
|  |  | Sample 22 | - | 0/1 | 71 | 0/1 | - | - | - | - |
|  |  | Sample 23 | - | 1/0 | 52 | 1/0 | - | - | - | - |
|  |  | Sample 24 | - | 0/1 | 76 | 0/1 | - | - | - | - |
