# Supplementary material for: Proteomic Analysis of Vitreous Humor in Retinal Vein Occlusion
Source: PLoS One. 2016 Jun 30;11(6):e0158001. doi: 10.1371/journal.pone.0158001 (PMC4928959; doi:10.1371/journal.pone.0158001)
Supplement: S2 Table — (DOCX) [file pone.0158001.s003.docx]

| **Table S2. 94 proteins being detected in vitreous humor**.  Proteins in vitreous humor detected by capillary electrophoresis coupled to mass spectrometer (CE-MS) and identified by tandem mass spectrometry (LC-MS/MS) when comparing protein signal intensity of retinal vein occlusion (RVO)-samples compared to control-samples. | | | | | | | | | | | | | |
| --- | --- | --- | --- | --- | --- | --- | --- | --- | --- | --- | --- | --- | --- |
| **Protein** | | **UniProt^‡^** | **RVO (n=30)** | | | | | **Control (n=16)** | | | | | ***P*-Value^§^** |
|  |  |  | Protein identification | | Statistical analysis | | | Protein identification | | Statistical analysis | | |  |
|  |  |  | Peptides^##^ | Coverage^†^ | N^#^ | Mean  intensity | SD | Peptides^##^ | Coverage^†^ | N^#^ | Mean intensity | SD |  |
| Afamin | | P43652 | 3 | 5 | 19 | 34.6 | 105.6 | 3 | 5 | 12 | 48.9 | 53.3 | 7.07E-02 |
| Alpha-1-acid glycoprotein 1 | | P02763 | 7 | 34 | 27 | 144.3 | 129.1 | 8 | 41 | 14 | 75.8 | 50.2 | 7.19E-02 |
| Alpha-1-acid glycoprotein 2 | | P19652 | 3 | 15 | 26 | 86.7 | 69.2 | 4 | 16 | 12 | 68.6 | 63.6 | 4.59E-01 |
| Alpha-1-antitrypsin | | P01009 | 23 | 57 | 30 | 287.6 | 146.1 | 23 | 62 | 16 | 272.4 | 101.1 | 8.00E-01 |
| Alpha-1B-glycoprotein | | P04217 | 2 | 4 | 6 | 9.4 | 27.2 | 3 | 7 | 4 | 5.7 | 14.7 | 8.73E-01 |
| Alpha-2-HS-glycoprotein | | P02765 | 3 | 13 | 11 | 5.7 | 10.2 | 3 | 10 | 8 | 10.3 | 16.5 | 3.02E-01 |
| Alpha-2-macroglobulin | | P01023 | 17 | 1 | 30 | 97.7 | 294.9 | 13 | 10 | 15 | 23.6 | 16.2 | 3.93E-01 |
| Alpha-crystallin B chain | | P02511 | 2 | 11 | 3 | 1.9 | 7.4 | 5 | 30 | 7 | 30.1 | 75.5 | 9.20E-03 |
| Amyloid-like protein 2 | | Q06481 | 7 | 11 | 14 | 9.1 | 27.2 | 5 | 8 | 9 | 5.6 | 9.4 | 7.67E-01 |
| Angiotensinogen | | P01019 | 2 | 5 | 10 | 15.0 | 33.7 | 2 | 5 | 8 | 49.8 | 83.5 | 1.29E-01 |
| Antithrombin-III | | P01008 | 11 | 27 | 28 | 71.1 | 33.1 | 11 | 27 | 14 | 50.2 | 31.7 | 5.55E-02 |
| Apolipoprotein A-I | | P02647 | 17 | 70 | 26 | 184.4 | 75.8 | 17 | 74 | 15 | 142.0 | 87.6 | 1.17E-01 |
| Apolipoprotein A-II | | P02652 | 2 | 29 | 30 | 8.1 | 14.4 | 4 | 41 | 15 | 50.1 | 67.6 | 1.78E-02 |
| Apolipoprotein A-IV | | P06727 | 19 | 56 | 10 | 47.0 | 35.6 | 16 | 51 | 10 | 147.4 | 352.9 | 8.36E-01 |
| Apolipoprotein E | | P02649 | 13 | 41 | 30 | 123.1 | 147.8 | 12 | 37 | 16 | 112.1 | 116.1 | 6.78E-01 |
| Beta-2-glycoprotein 1 | | P02749 | 2 | 7 | 4 | 0.5 | 1.3 | 1 | 3 | 4 | 4.6 | 12.8 | 2.09E-01 |
| Beta-crystallin B2 | | P43320 | 6 | 42 | 11 | 18.3 | 35.3 | 7 | 40 | 5 | 12.0 | 39.7 | 6.16E-01 |
| Calmodulin-regulated spectrin-associated protein 3**^§§^** | | Q9P1Y5 | 1 | 1 | 2 | 42.7 | 165.7 | 2 | 2 | 3 | 1.6 | 4.9 | 2.67E-01 |
| Cartilage glycoprotein-39**^§§^** | | Q9NY41 | 2 | 13 | 12 | 3.0 | 4.9 | 3 | 20 | 7 | 6.9 | 12.6 | 5.70E-01 |
| Cathepsin D | | P07339 | 6 | 20 | 29 | 242.1 | 467.3 | 7 | 22 | 15 | 553.9 | 1198.1 | 7.38E-01 |
| Ceruloplasmin | | P00450 | 15 | 22 | 30 | 207.4 | 180.6 | 17 | 21 | 15 | 157.1 | 187.6 | 9.68E-02 |
| Clusterin* | | P10909 | 9 | 26 | 30 | 524.1 | 194.2 | 10 | 28 | 15 | 282.3 | 174.6 | 4.97E-04 |
| Collagen alpha-1(I) chain | | P02452 | 4 | 4 | 14 | 7.7 | 10.2 | 4 | 2 | 4 | 5.1 | 11.3 | 2.18E-01 |
| Collagen alpha-1(II) chain | | P02458 | 29 | 25 | 30 | 95.7 | 132.7 | 35 | 45 | 15 | 371.1 | 1026.1 | 2.99E-01 |
| Collagen alpha-1(III) chain | | P02461 | 3 | 4 | 23 | 93.4 | 91.6 | 3 | 4 | 10 | 37.9 | 41.8 | 4.48E-02 |
| Collagen alpha-1(V) chain | | P20908 | 5 | 4 | 22 | 35.2 | 36.8 | 6 | 4 | 13 | 64.7 | 142.9 | 7.10E-01 |
| Collagen alpha-1(VII) chain | | Q02388 | 3 | 2 | 5 | 29.3 | 101.6 | 2 | 1 | 8 | 25.1 | 57.2 | 2.94E-02 |
| Collagen alpha-1(IX) chain | | P20849 | 9 | 15 | 21 | 18.9 | 32.3 | 9 | 16 | 12 | 73.1 | 187.1 | 8.16E-01 |
| Collagen alpha-1(XI) chain | | P12107 | 5 | 4 | 14 | 88.2 | 317.3 | 6 | 4 | 8 | 145.8 | 444.7 | 9.80E-01 |
| Collagen alpha-1(XII) chain | | Q99715 | 2 | 2 | 14 | 13.3 | 19.2 | 2 | 2 | 5 | 6.1 | 14.5 | 2.06E-01 |
| Collagen alpha-1(XXII) chain | | Q8NFW1 | 6 | 6 | 18 | 24.0 | 35.6 | 6 | 6 | 9 | 14.0 | 22.0 | 4.88E-01 |
| Collagen alpha-1(XXIV) chain**^§§^** | | Q17RW2 | 1 | 1 | 1 | 1.9 | 10.5 | 2 | 2 | 3 | 6.6 | 20.7 | 8.50E-02 |
| Collagen alpha-1(XXVII) chain**^§§^** | | Q8IZC6 | 2 | 3 | 4 | 1.2 | 3.8 | 1 | 2 | 2 | 257.3 | 854.5 | 9.37E-01 |
| Collagen alpha-1(XXVIII) chain | | Q2UY09 | 1 | 1 | 1 | 2.0 | 11.1 | 1 | 2 | 1 | 16.9 | 67.5 | 6.25E-01 |
| Collagen alpha-2(I) chain | | P08123 | 4 | 7 | 18 | 15.1 | 15.8 | 4 | 7 | 6 | 5.1 | 8.1 | 4.32E-02 |
| Collagen alpha-2(IX) chain | | Q14055 | 3 | 6 | 6 | 17.2 | 87.5 | 5 | 9 | 5 | 134.1 | 443.6 | 2.95E-01 |
| Collagen alpha-2(XI) chain | | P13942 | 3 | 3 | 30 | 740.4 | 678.7 | 4 | 3 | 13 | 377.1 | 528.5 | 8.55E-03 |
| Collagen alpha-3(IX) chain | | Q14050 | 8 | 15 | 18 | 64.6 | 104.3 | 8 | 17 | 11 | 155.8 | 156.3 | 8.41E-02 |
| Collagen alpha-4(IV) chain**^§§^** | | P53420 | 1 | 1 | 1 | 1.3 | 7.1 | 1 | 1 | 2 | 2.0 | 5.6 | 2.58E-01 |
| Collagen alpha-5(IV) chain | | P29400 | 2 | 2 | 24 | 251.2 | 271.9 | 2 | 2 | 16 | 264.0 | 353.1 | 8.35E-01 |
| Complement C3* | | P01024 | 43 | 29 | 30 | 582.3 | 134.3 | 41 | 27 | 16 | 390.2 | 194.5 | 1.85E-03 |
| Complement C4-A | | P0C0L4 | **2** | 2 | 4 | 2.0 | 6.9 | 4 | 3 | 7 | 29.3 | 78.3 | 1.91E-02 |
| Complement component 4B preproprotein | | P0C0L5 | 13 | 10 | 26 | 18.0 | 17.0 | 12 | 10 | 13 | 18.2 | 23.0 | 5.48E-01 |
| Complement component C9 | | P02748 | 2 | 4 | 28 | 280.4 | 175.9 | 2 | 4 | 13 | 245.2 | 194.7 | 7.12E-01 |
| Complement factor B | | P00751 | 2 | 3 | 13 | 18.1 | 60.4 | 4 | 6 | 11 | 44.9 | 61.4 | 1.23E-02 |
| Cystatin-C | | P01034 | 4 | 30 | 25 | 48.4 | 90.8 | 3 | 23 | 12 | 39.9 | 38.3 | 5.78E-01 |
| Dickkopf-related protein 3 | | Q9UBP4 | 6 | 22 | 27 | 92.7 | 102.6 | 6 | 27 | 14 | 129.9 | 142.7 | 3.21E-01 |
| Fibrinogen alpha chain | | P02671 | 4 | 8 | 16 | 8.9 | 11.2 | 4 | 8 | 6 | 12.0 | 31.1 | 3.70E-01 |
| Fibrinogen beta chain | | P02675 | 3 | 6 | 18 | 27.3 | 38.3 | 4 | 9 | 7 | 6.9 | 14.1 | 6.54E-02 |
| Fizzy-related protein homolog**^§§^** | | Q9UM11 | 2 | 5 | 12 | 5.8 | 9.9 | 1 | 1 | 6 | 2.7 | 4.4 | 4.96E-01 |
| Gelsolin | | P06396 | 4 | 6 | 17 | 26.2 | 43.8 | 3 | 5 | 12 | 29.5 | 66.1 | 9.25E-01 |
| Glutathione peroxidase 3 | | P22352 | 4 | 18 | 24 | 88.4 | 83.7 | 4 | 18 | 12 | 78.7 | 174.6 | 1.57E-01 |
| Haptoglobin | | P00738 | 8 | 28 | 24 | 49.5 | 92.3 | 9 | 24 | 10 | 30.6 | 51.3 | 2.74E-01 |
| Hemoglobin subunit beta | | P68871 | 4 | 36 | 14 | 94.8 | 389.5 | 3 | 34 | 7 | 38.6 | 136.9 | 3.65E-01 |
| Hemopexin | | P02790 | 14 | 34 | 30 | 133.7 | 55.5 | 12 | 28 | 15 | 159.3 | 92.7 | 1.74E-01 |
| Heparin cofactor 2 | | P05546 | 1 | 2 | 4 | 1.3 | 4.5 | 2 | 4 | 4 | 1.3 | 3.0 | 3.64E-01 |
| Histidine-rich glycoprotein | | P04196 | 2 | 4 | 5 | 7.9 | 26.8 | 1 | 2 | 4 | 6.9 | 17.7 | 5.71E-01 |
| Ig alpha-1 chain C region | | P01876 | 2 | 4 | 13 | 40.8 | 88.0 | 2 | 4 | 5 | 16.0 | 41.0 | 3.26E-01 |
| Ig alpha-2 chain C region | | P01877 | 4 | 15 | 25 | 132.3 | 144.8 | 2 | 6 | 11 | 84.2 | 88.9 | 2.33E-01 |
| Ig gamma-1 chain C region | | P01857 | 11 | 32 | 30 | 787.7 | 358.7 | 11 | 32 | 16 | 725.9 | 462.5 | 4.20E-01 |
| Ig gamma-2 chain C region | | P01859 | 2 | 6 | 21 | 35.2 | 41.8 | 2 | 6 | 14 | 65.0 | 94.3 | 3.07E-01 |
| Ig gamma-3 chain C region | | P01860 | 3 | 10 | 12 | 44.7 | 87.8 | 4 | 12 | 8 | 47.2 | 94.0 | 6.65E-01 |
| Ig heavy chain V-III region GAL | | P01781 | 3 | 27 | 11 | 11.3 | 24.1 | 2 | 17 | 7 | 40.3 | 65.3 | 3.01E-01 |
| Ig kappa chain C region | | P01834 | 4 | 80 | 27 | 113.8 | 116.6 | 4 | 80 | 11 | 110.8 | 108.3 | 8.81E-01 |
| Ig kappa chain V-I region EU | | P01598 | 2 | 27 | 4 | 7.1 | 29.6 | 1 | 17 | 3 | 3.1 | 7.4 | 6.58E-01 |
| Ig lambda-2 chain C regions | | P0CG05 | 2 | 27 | 25 | 717.5 | 525.6 | 3 | 42 | 12 | 452.7 | 305.4 | 7.85E-02 |
| IgGFc-binding protein | | Q9Y6R7 | 4 | 1 | 20 | 117.2 | 309.7 | 4 | 1 | 14 | 181.5 | 268.0 | 1.63E-01 |
| Ig lambda-like polypeptide 5* | | B9A064 | 3 | 20 | 30 | 358.2 | 422.5 | 2 | 13 | 11 | 93.4 | 75.7 | 1.43E-05 |
| Inter-alpha (Globulin) inhibitor H2 | | A2RTY6 | 2 | 2 | 18 | 18.9 | 23.9 | 2 | 2 | 6 | 12.5 | 26.5 | 1.71E-01 |
| Inter-alpha-trypsin inhibitor heavy chain H1 | | P19827 | 3 | 4 | 28 | 107.9 | 128.4 | 3 | 4 | 13 | 92.5 | 79.6 | 9.45E-01 |
| Inter-alpha-trypsin inhibitor heavy chain H4 | | Q14624 | 2 | 2 | 8 | 4.2 | 9.7 | 1 | 1 | 1 | 0.0 | 0.2 | 7.76E-02 |
| Kininogen-1 | | P01042 | 5 | 9 | 9 | 4.9 | 8.9 | 6 | 11 | 7 | 18.4 | 47.8 | 3.49E-01 |
| Leucine-rich alpha-2-glycoprotein | | P02750 | 1 | 3 | 8 | 26.5 | 86.4 | 2 | 6 | 7 | 25.8 | 47.6 | 3.19E-01 |
| Neuroblast differentiation-associated protein AHNAK**^§§^** | | Q09666 | 2 | 1 | 6 | 1.4 | 3.9 | 4 | 1 | 9 | 14.0 | 24.8 | 3.34E-03 |
| Obscurin | | Q5VST9 | 2 | 0 | 21 | 520.3 | 1358.1 | 2 | 0 | 16 | 1767.1 | 3857.7 | 1.51E-02 |
| Opticin* | | Q9UBM4 | 6 | 20 | 23 | 82.8 | 207.2 | 5 | 16 | 16 | 163.7 | 95.1 | 7.07E-05 |
| Osteopontin | | P10451 | 5 | 28 | 25 | 78.7 | 140.7 | 4 | 18 | 13 | 53.1 | 80.0 | 7.37E-01 |
| Pigment epithelium-derivedfactor | | P36955 | 12 | 31 | 30 | 185.7 | 75.7 | 15 | 36 | 15 | 161.2 | 134.1 | 1.59E-01 |
| Plasminogen | | P00747 | 2 | 2 | 9 | 3.9 | 7.3 | 2 | 2 | 7 | 12.8 | 18.6 | 1.62E-01 |
| Prostaglandin-H2 D-isomerase | | P41222 | 4 | 21 | 27 | 2589.7 | 2630.4 | 4 | 21 | 13 | 4122.9 | 6744.6 | 1.00E+00 |
| Protein Jade-2 | | Q9NQC1 | 1 | 1 | 1 | 0.1 | 0.6 | 2 | 3 | 2 | 0.2 | 0.7 | 2.58E-01 |
| Protein S100-A9 | | P06702 | 1 | 6 | 3 | 0.2 | 0.6 | 2 | 18 | 5 | 44.6 | 155.8 | 7.79E-02 |
| Prothrombin | | P00734 | 2 | 4 | 8 | 14.8 | 32.7 | 2 | 4 | 3 | 25.6 | 88.9 | 5.17E-01 |
| Retinol-binding protein 3 | | P10745 | 19 | 22 | 30 | 254.7 | 514.0 | 15 | 17 | 16 | 605.1 | 1113.3 | 6.50E-02 |
| Serine/cysteine proteinase inhibitor clade G member 1 splice variant 2**^§§^** | | Q5UGI6 | 5 | 19 | 19 | 20.9 | 32.4 | 6 | 20 | 12 | 18.0 | 30.6 | 9.63E-01 |
| Serotransferrin | | P02787 | 38 | 49 | 30 | 264.5 | 82.0 | 33 | 45 | 16 | 323.7 | 181.1 | 6.61E-01 |
| SERPINA3 Alpha-1-antichymotrypsin | | P01011 | 14 | 31 | 30 | 44.9 | 26.8 | 13 | 31 | 16 | 43.1 | 30.3 | 8.54E-01 |
| Serum albumin | | P02768 | 40 | 61 | 30 | 2109.7 | 657.6 | 42 | 65 | 16 | 2910.3 | 1163.1 | 2.11E-02 |
| Titin | | Q8WZ42 | 3 | <1 | 16 | 6.1 | 8.0 | 4 | <1 | 6 | 3.5 | 6.1 | 2.96E-01 |
| Translational activator GCN1**^§§^** | | Q92616 | 2 | 1 | 2 | 184.9 | 340.9 | 2 | 1 | 1 | 62.8 | 122.1 | 5.01E-01 |
| Transthyretin | | P02766 | 7 | 69 | 27 | 276.6 | 247.7 | 7 | 50 | 14 | 517.6 | 479.2 | 1.52E-01 |
| Vitamin D-binding protein | | **P02774** | 8 | 14 | 22 | 22.1 | 27.6 | 8 | 15 | 11 | 25.3 | 32.1 | 9.07E-01 |
| Vitronectin* | | P04004 | 4 | 10 | 25 | 89.6 | 136.0 | 3 | 8 | 8 | 8.8 | 11.0 | 2.74E-04 |
| Zinc-alpha-2-glycoprotein | | **P25311** | 3 | 14 | 19 | 37.7 | 55.8 | 3 | 14 | 7 | 12.5 | 24.5 | 9.43E-02 |
|  | |  |  |  |  |  |  |  |  |  |  |  |  |
| ‡  ##  †  #  §  *  §§ | Listed in the universal protein resource (UniProt), a central repository of protein data.  Number of peptides observed by CE-MS analysis and sequenced by LC-MS/MS for each protein.  Percentage (%) of peptide coverage of the protein sequence.  Number of samples with a signal intensity >0  *P*-Value was analyzed by using the Mann-Whitney test. A *P* of α<5.00E-02 was considered statistically significant. Significant proteins are highlighted in light grey.  Proteins which remained significant after performing multiple hypotheses testing correction, analyzed by using the Benjamini-Hochberg test for false discovery rate (see Table 2). A adjusted *P*-Value of α<5.00E-02 was considered statistically significant.  Proteins being not described in previous studies of human vitreous humor proteome [12, 38-40]. | | | | | | | | | | | | |
