# Supplementary material for: Proteomic Analysis of Vitreous Humor in Retinal Vein Occlusion
Source: PLoS One. 2016 Jun 30;11(6):e0158001. doi: 10.1371/journal.pone.0158001 (PMC4928959; doi:10.1371/journal.pone.0158001)
Supplement: S4 Table — (DOCX) [file pone.0158001.s005.docx]

Table S4. Subgroup analysis – detailed information.

Proteins in vitreous humor detected by capillary electrophoresis coupled to mass spectrometer (CE-MS) and identified by tandem mass spectrometry (LC-MS/MS) when comparing protein signal intensity of subgroups of retinal vein occlusion (RVO)-samples (central RVO (CRVO), hemi-central RVO (H-CRVO), branch RVO (BRVO)) compared to control-samples.

| **Protein** | | | **UniPort^‡^** | **Control (n=16)** | | | **CRVO (n=14)** | | | ***P*-Value^§^**  Control  vs.  CRVO | **H-CRVO (n=6)** | | | | ***P*-Value^§^**  Control  vs.  H-CRVO | | **BRVO (n=10)** | | | | | ***P*-Value^§^**  Control  vs.  BRVO | |
| --- | --- | --- | --- | --- | --- | --- | --- | --- | --- | --- | --- | --- | --- | --- | --- | --- | --- | --- | --- | --- | --- | --- | --- |
|  |  |  |  | N^#^ | Signal intensity | | N^#^ | Signal intensity | |  | N^#^ | Signal intensity | | |  |  | N^#^ | | Signal intensity | | |  |  |
|  |  |  |  |  | Mean | SD |  | Mean | SD |  |  | Mean | SD | |  |  |  |  | Mean | SD | |  |  |
| Afamin | | | P43652 | 12 | 48.9 | 53.3 | 11 | 61.1 | 152.1 | 4.51E-01 | 3 | 11.4 | 14.9 | | 9.93E-02 | | 5 | | 11.3 | 18.3 | | 4.64E-02 | |
| Alpha-1-acid glycoprotein 1 | | | P02763 | 14 | 75.8 | 50.2 | 13 | 150.5 | 117.2 | 3.76E-02 | 5 | 195.8 | 180.2 | | 1.40E-01 | | 9 | | 104.6 | 110.6 | | 6.73E-01 | |
| Alpha-1-acid glycoprotein 2 | | | P19652 | 12 | 68.6 | 63.6 | 13 | 108.8 | 78.7 | 1.97E-01 | 5 | 80.3 | 56.3 | | 6.04E-01 | | 8 | | 59.7 | 55.7 | | 7.50E-01 | |
| Alpha-1-antitrypsin | | | P01009 | 16 | 272.4 | 101.1 | 14 | 249.3 | 99.1 | 4.30E-01 | 6 | 273.1 | 74.4 | | 1.00E+00 | | 10 | | 350.0 | 211.6 | | 6.73E-01 | |
| Alpha-1B-glycoprotein | | | P04217 | 4 | 5.7 | 14.7 | 1 | 9.3 | 34.9 | 2.49E-01 | 2 | 5.9 | 10.7 | | 6.39E-01 | | 3 | | 11.5 | 23.4 | | 5.89E-01 | |
| Alpha-2-HS-glycoprotein | | | P02765 | 8 | 10.3 | 16.5 | 5 | 5.0 | 7.2 | 4.08E-01 | 2 | 1.8 | 2.7 | | 1.98E-01 | | 4 | | 9.1 | 15.1 | | 7.31E-01 | |
| Alpha-2-macroglobulin | | | P01023 | 15 | 23.6 | 16.2 | 14 | 59.3 | 129.4 | 1.00E+00 | 6 | 290.9 | 634.7 | | 2.69E-01 | | 10 | | 35.6 | 25.5 | | 2.46E-01 | |
| Alpha-crystallin B chain | | | P02511 | 7 | 30.1 | 75.5 | 2 | 3.2 | 10.4 | 9.07E-02 | 0 | 0.0 | 0.0 | | 6.12E-02 | | 1 | | 1.2 | 3.8 | | 7.63E-02 | |
| Amyloid-like protein 2 | | | Q06481 | 9 | 5.6 | 9.4 | 6 | 4.2 | 7.8 | 5.05E-01 | 3 | 26.6 | 59.7 | | 9.08E-01 | | 5 | | 5.6 | 7.3 | | 9.34E-01 | |
| Angiotensinogen | | | P01019 | 8 | 49.8 | 83.5 | 7 | 12.6 | 16.1 | 4.77E-01 | 1 | 2.5 | 6.2 | | 9.81E-02 | | 2 | | 26.0 | 54.9 | | 1.86E-01 | |
| Antithrombin-III | | | P01008 | 14 | 50.2 | 31.7 | 12 | 58.9 | 35.5 | 4.54E-01 | 6 | 84.4 | 32.6 | | 6.52E-02 | | 10 | | 80.2 | 26.1 | | 3.50E-02 | |
| Apolipoprotein A-I | | | P02647 | 15 | 142.0 | 87.6 | 14 | 165.7 | 89.8 | 5.06E-01 | 6 | 209.7 | 68.1 | | 1.61E-01 | | 10 | | 195.5 | 56.4 | | 8.20E-02 | |
| Apolipoprotein A-II | | | P02652 | 10 | 50.1 | 67.6 | 4 | 10.0 | 16.8 | 4.23E-02 | 2 | 3.2 | 5.1 | | 1.22E-01 | | 4 | | 8.5 | 15.1 | | 1.20E-01 | |
| Apolipoprotein A-IV | | | P06727 | 16 | 147.4 | 352.9 | 14 | 35.8 | 35.9 | 2.80E-01 | 6 | 47.8 | 25.1 | | 7.68E-01 | | 10 | | 62.1 | 37.7 | | 4.93E-01 | |
| Apolipoprotein E | | | P02649 | 15 | 112.1 | 116.1 | 11 | 66.4 | 124.7 | 5.83E-02 | 5 | 136.5 | 152.9 | | 9.71E-01 | | 10 | | 194.4 | 155.6 | | 1.55E-01 | |
| Beta-2-glycoprotein 1 | | | P02749 | 4 | 4.6 | 12.8 | 3 | 0.8 | 1.7 | 5.75E-01 | 1 | 0.6 | 1.4 | | 5.47E-01 | | 0 | | 0.0 | 0.0 | | 9.33E-02 | |
| Beta-crystallin B2 | | | P43320 | 5 | 12.0 | 39.7 | 6 | 22.9 | 40.1 | 3.61E-01 | 2 | 9.6 | 23.0 | | 1.00E+00 | | 3 | | 17.2 | 36.2 | | 9.74E-01 | |
| Calmodulin-regulated spectrin-associated protein 3 | | | Q9P1Y5 | 3 | 1.6 | 4.9 | 0 | 0.0 | 0.0 | 9.37E-02 | 1 | 127.7 | 312.8 | | 9.56E-01 | | 1 | | 51.3 | 162.4 | | 6.44E-01 | |
| Cartilage glycoprotein-39 | | | Q9NY41 | 7 | 6.9 | 12.6 | 4 | 1.8 | 3.3 | 3.12E-01 | 3 | 2.6 | 3.4 | | 8.40E-01 | | 5 | | 4.9 | 6.9 | | 9.31E-01 | |
| Cathepsin D | | | P07339 | 15 | 553.9 | 1198.1 | 13 | 385.4 | 657.4 | 9.50E-01 | 6 | 64.4 | 52.2 | | 2.10E-01 | | 10 | | 148.0 | 122.4 | | 9.58E-01 | |
| Ceruloplasmin | | | P00450 | 15 | 157.1 | 187.6 | 14 | 272.6 | 242.8 | 1.14E-01 | 6 | 151.9 | 78.8 | | 3.02E-01 | | 10 | | 149.3 | 63.9 | | 2.92E-01 | |
| Clusterin*^; †; ††; †††; ††††^ | | | P10909 | 15 | 282.3 | 174.6 | 14 | 487.5 | 187.6 | 1.12E-02 | 6 | 570.4 | 248.9 | | 9.87E-03 | | 10 | | 547.6 | 179.5 | | 3.75E-03 | |
| Collagen alpha-1(I) chain | | | P02452 | 4 | 5.1 | 11.3 | 4 | 5.8 | 10.6 | 8.31E-01 | 4 | 8.9 | 8.0 | | 1.46E-01 | | 6 | | 9.6 | 11.3 | | 1.18E-01 | |
| Collagen alpha-1(II) chain | | | P02458 | 15 | 371.1 | 1026.1 | 14 | 121.8 | 188.8 | 7.39E-01 | 6 | 57.7 | 44.4 | | 7.12E-01 | | 10 | | 81.8 | 43.9 | | 9.17E-02 | |
| Collagen alpha-1(III) chain | | | P02461 | 10 | 37.9 | 41.8 | 10 | 106.7 | 108.8 | 9.86E-02 | 5 | 60.0 | 62.8 | | 4.10E-01 | | 8 | | 94.8 | 81.8 | | 5.42E-02 | |
| Collagen alpha-1(V) chain^†; †††^ | | | P20908 | 13 | 64.7 | 142.9 | 8 | 14.3 | 24.0 | 1.09E-01 | 5 | 34.8 | 23.2 | | 3.55E-01 | | 9 | | 64.7 | 39.9 | | 2.85E-02 | |
| Collagen alpha-1(VII) chain | | | Q02388 | 8 | 25.1 | 57.2 | 2 | 32.8 | 106.4 | 9.21E-02 | 2 | 2.8 | 5.4 | | 2.95E-01 | | 1 | | 40.3 | 127.5 | | 8.22E-02 | |
| Collagen alpha-1(IX) chain | | | P20849 | 12 | 73.1 | 187.1 | 9 | 18.7 | 42.1 | 7.05E-01 | 4 | 29.0 | 31.7 | | 5.51E-01 | | 8 | | 13.2 | 11.6 | | 5.60E-01 | |
| Collagen alpha-1(XI) chain | | | P12107 | 8 | 145.8 | 444.7 | 4 | 182.9 | 454.4 | 3.98E-01 | 4 | 4.5 | 5.6 | | 5.36E-01 | | 6 | | 5.9 | 7.1 | | 5.42E-01 | |
| Collagen alpha-1(XII) chain | | | Q99715 | 5 | 6.1 | 14.5 | 5 | 6.6 | 10.5 | 7.47E-01 | 3 | 7.6 | 8.7 | | 3.69E-01 | | 6 | | 26.1 | 26.7 | | 6.07E-02 | |
| Collagen alpha-1(XXII) chain | | | Q8NFW1 | 9 | 14.0 | 22.0 | 7 | 28.5 | 44.9 | 7.76E-01 | 5 | 22.7 | 27.5 | | 2.42E-01 | | 6 | | 18.6 | 26.4 | | 7.01E-01 | |
| Collagen alpha-1(XXIV) chain | | | Q17RW2 | 3 | 6.6 | 20.7 | 0 | 0.0 | 0.0 | 9.37E-02 | 1 | 9.5 | 23.4 | | 9.56E-01 | | 0 | | 0.0 | 0.0 | | 1.54E-01 | |
| Collagen alpha-1(XXVII) chain | | | Q8IZC6 | 2 | 257.3 | 854.5 | 1 | 1.2 | 4.7 | 5.76E-01 | 3 | 3.0 | 4.6 | | 1.32E-01 | | 0 | | 0.0 | 0.0 | | 2.54E-01 | |
| Collagen alpha-1(XXVIII) chain | | | Q2UY09 | 1 | 16.9 | 67.5 | 0 | 0.0 | 0.0 | 3.50E-01 | 1 | 10.1 | 24.7 | | 5.06E-01 | | 0 | | 0.0 | 0.0 | | 4.29E-01 | |
| Collagen alpha-2(I) chain | | | P08123 | 6 | 5.1 | 8.1 | 7 | 13.9 | 19.3 | 3.73E-01 | 4 | 16.9 | 14.5 | | 6.41E-02 | | 7 | | 15.6 | 11.9 | | 2.43E-02 | |
| Collagen alpha-2(IX) chain | | | Q14055 | 5 | 134.1 | 443.6 | 2 | 34.7 | 128.2 | 2.87E-01 | 3 | 3.6 | 7.4 | | 7.65E-01 | | 1 | | 1.0 | 3.1 | | 1.87E-01 | |
| Collagen alpha-2(XI) chain^†; ††; †††^ | | | P13942 | 13 | 377.1 | 528.5 | 14 | 1097.0 | 856.5 | 9.92E-03 | 6 | 371.7 | 40.9 | | 1.84E-01 | | 10 | | 462.3 | 222.2 | | 7.29E-02 | |
| Collagen alpha-3(IX) chain | | | Q14050 | 11 | 155.8 | 156.3 | 10 | 101.0 | 135.5 | 4.48E-01 | 1 | 30.9 | 75.6 | | 4.84E-02 | | 7 | | 33.9 | 38.7 | | 1.15E-01 | |
| Collagen alpha-4(IV) chain | | | P53420 | 2 | 2.0 | 5.6 | 0 | 0.0 | 0.0 | 1.78E-01 | 1 | 6.5 | 15.9 | | 7.11E-01 | | 0 | | 0.0 | 0.0 | | 2.54E-01 | |
| Collagen alpha-5(IV) chain | | | P29400 | 16 | 264.0 | 353.1 | 12 | 258.9 | 284.9 | 7.71E-01 | 4 | 150.9 | 119.8 | | 5.55E-01 | | 8 | | 300.5 | 321.6 | | 7.52E-01 | |
| Complement C3*^; †; ††; †††; ††††^ | | | P01024 | 16 | 390.2 | 194.5 | 14 | 569.1 | 167.6 | 1.99E-02 | 6 | 603.5 | 84.2 | | 1.83E-02 | | 10 | | 588.1 | 113.9 | | 1.32E-02 | |
| Complement C4-A^†; ††; †††^ | | | P0C0L4 | 7 | 29.3 | 78.3 | 0 | 0.0 | 0.0 | 6.01E-03 | 2 | 7.5 | 14.6 | | 6.79E-01 | | 2 | | 1.5 | 3.3 | | 1.72E-01 | |
| Complement component 4B preproprotein | | | P0C0L5 | 13 | 18.2 | 23.0 | 12 | 16.7 | 17.1 | 7.71E-01 | 5 | 17.4 | 11.5 | | 6.84E-01 | | 9 | | 20.3 | 20.6 | | 4.76E-01 | |
| Complement component C9 | | | P02748 | 13 | 245.2 | 194.7 | 12 | 225.3 | 147.5 | 8.35E-01 | 6 | 273.6 | 130.6 | | 8.83E-01 | | 10 | | 361.5 | 216.3 | | 3.16E-01 | |
| Complement factor B^†; ††; ††††^ | | | P00751 | 11 | 44.9 | 61.4 | 8 | 13.3 | 21.2 | 9.64E-02 | 1 | 1.2 | 3.0 | | 1.82E-02 | | 4 | | 35.1 | 102.7 | | 7.05E-02 | |
| Cystatin-C | | | P01034 | 12 | 39.9 | 38.3 | 13 | 75.3 | 126.4 | 9.01E-01 | 6 | 14.2 | 9.5 | | 2.37E-01 | | 6 | | 31.2 | 35.0 | | 5.56E-01 | |
| Dickkopf-related protein 3 | | | Q9UBP4 | 14 | 129.9 | 142.7 | 12 | 94.1 | 134.8 | 1.97E-01 | 5 | 45.0 | 31.6 | | 1.21E-01 | | 10 | | 119.3 | 69.2 | | 6.35E-01 | |
| Fibrinogen alpha chain^†; ††; ††††^ | | | P02671 | 6 | 12.0 | 31.1 | 6 | 6.3 | 8.7 | 7.78E-01 | 6 | 18.7 | 9.9 | | 2.02E-02 | | 4 | | 6.8 | 12.7 | | 1.00E+00 | |
| Fibrinogen beta chain | | | P02675 | 7 | 6.9 | 14.1 | 9 | 25.3 | 41.0 | 8.33E-02 | 4 | 15.0 | 13.5 | | 2.08E-01 | | 5 | | 37.6 | 44.4 | | 2.40E-01 | |
| Fizzy-related protein homolog | | | Q9UM11 | 6 | 2.7 | 4.4 | 7 | 6.2 | 7.3 | 2.15E-01 | 3 | 9.7 | 18.0 | | 5.08E-01 | | 2 | | 2.8 | 6.0 | | 5.62E-01 | |
| Gelsolin | | | P06396 | 12 | 29.5 | 66.1 | 10 | 20.8 | 19.4 | 6.44E-01 | 4 | 55.0 | 80.9 | | 3.72E-01 | | 3 | | 16.3 | 36.2 | | 1.39E-01 | |
| Glutathione peroxidase 3 | | | P22352 | 12 | 78.7 | 174.6 | 10 | 60.1 | 86.8 | 9.33E-01 | 5 | 92.4 | 76.2 | | 2.66E-01 | | 9 | | 125.7 | 75.1 | | 2.00E-02 | |
| Haptoglobin^†; †††; ††††^ | | | P00738 | 10 | 30.6 | 51.3 | 9 | 14.9 | 23.8 | 7.33E-01 | 5 | 24.3 | 21.1 | | 5.00E-01 | | 10 | | 113.0 | 140.1 | | 1.47E-02 | |
| Hemoglobin subunit beta | | | P68871 | 7 | 38.6 | 136.9 | 5 | 168.5 | 570.8 | 8.33E-01 | 1 | 14.2 | 34.7 | | 3.69E-01 | | 8 | | 40.1 | 35.7 | | 2.13E-02 | |
| Hemopexin | | | P02790 | 15 | 159.3 | 92.7 | 14 | 145.4 | 61.0 | 3.83E-01 | 6 | 135.9 | 60.2 | | 5.07E-01 | | 10 | | 116.0 | 44.2 | | 1.26E-01 | |
| Heparin cofactor 2 | | | P05546 | 4 | 1.3 | 3.0 | 1 | 0.2 | 0.7 | 1.79E-01 | 2 | 2.2 | 3.5 | | 6.39E-01 | | 1 | | 2.3 | 7.3 | | 4.44E-01 | |
| Histidine-rich glycoprotein | | | P04196 | 4 | 6.9 | 17.7 | 2 | 9.8 | 35.1 | 5.13E-01 | 1 | 11.5 | 28.3 | | 8.41E-01 | | 2 | | 3.1 | 6.9 | | 7.75E-01 | |
| Ig alpha-1 chain C region | | | P01876 | 5 | 16.0 | 41.0 | 4 | 11.5 | 29.5 | 8.78E-01 | 5 | 84.8 | 113.4 | | 1.76E-02 | | 4 | | 55.4 | 117.0 | | 4.95E-01 | |
| Ig alpha-2 chain C region | | | P01877 | 11 | 84.2 | 88.9 | 10 | 123.5 | 199.5 | 8.33E-01 | 5 | 139.8 | 101.9 | | 1.93E-01 | | 10 | | 140.3 | 64.0 | | 3.92E-02 | |
| Ig gamma-1 chain C region | | | P01857 | 16 | 725.9 | 462.5 | 14 | 683.4 | 432.6 | 8.35E-01 | 6 | 826.6 | 234.5 | | 4.61E-01 | | 10 | | 910.4 | 281.2 | | 1.14E-01 | |
| Ig gamma-2 chain C region | | | P01859 | 14 | 65.0 | 94.3 | 10 | 46.5 | 50.5 | 8.02E-01 | 3 | 7.8 | 11.7 | | 2.61E-02 | | 8 | | 35.7 | 34.2 | | 6.73E-01 | |
| Ig gamma-3 chain C region | | | P01860 | 8 | 47.2 | 94.0 | 6 | 59.9 | 110.1 | 9.64E-01 | 3 | 30.0 | 64.6 | | 8.13E-01 | | 3 | | 32.3 | 65.9 | | 4.46E-01 | |
| Ig heavy chain V-III region GAL | | | P01781 | 7 | 40.3 | 65.3 | 6 | 17.8 | 33.0 | 5.81E-01 | 1 | 4.7 | 11.6 | | 2.15E-01 | | 4 | | 6.2 | 10.1 | | 4.46E-01 | |
| Ig kappa chain C region | | | P01834 | 11 | 110.8 | 108.3 | 11 | 98.5 | 125.9 | 6.29E-01 | 6 | 155.3 | 156.1 | | 4.15E-01 | | 10 | | 110.3 | 76.6 | | 7.11E-01 | |
| Ig kappa chain V-I region EU | | | P01598 | 3 | 3.1 | 7.4 | 3 | 14.6 | 42.8 | 7.44E-01 | 1 | 1.4 | 3.4 | | 7.84E-01 | | 0 | | 0.0 | 0.0 | | 1.54E-01 | |
| Ig lambda-2 chain C regions^†; †††; ††††^ | | | P0CG05 | 12 | 452.7 | 305.4 | 10 | 484.6 | 494.0 | 9.67E-01 | 5 | 684.3 | 465.2 | | 2.36E-01 | | 10 | | 1063.4 | 446.5 | | 1.84E-03 | |
| IgGFc-binding protein^†; ††; ††††^ | | | Q9Y6R7 | 14 | 181.5 | 268.0 | 9 | 170.1 | 443.2 | 1.22E-01 | 2 | 11.3 | 17.7 | | 2.10E-02 | | 9 | | 106.6 | 115.0 | | 6.73E-01 | |
| Ig lambda-like polypeptide 5*^; †; ††; †††; ††††^ | | | B9A064 | 11 | 93.4 | 75.7 | 14 | 215.4 | 118.0 | 2.05E-03 | 6 | 561.9 | 619.6 | | 1.11E-03 | | 10 | | 435.9 | 525.0 | | 2.14E-04 | |
| Inter-alpha (Globulin) inhibitor H2 | | | A2RTY6 | 6 | 12.5 | 26.5 | 8 | 13.6 | 22.1 | 5.28E-01 | 3 | 17.1 | 22.2 | | 5.63E-01 | | 7 | | 27.4 | 27.0 | | 4.87E-02 | |
| Inter-alpha-trypsin inhibitor heavy chain H1 | | | P19827 | 13 | 92.5 | 79.6 | 13 | 126.6 | 165.0 | 9.83E-01 | 5 | 82.0 | 119.9 | | 3.55E-01 | | 10 | | 97.3 | 68.0 | | 5.98E-01 | |
| Inter-alpha-trypsin inhibitor heavy chain H4 | | | Q14624 | 1 | 0.0 | 0.2 | 5 | 6.8 | 13.2 | 3.47E-02 | 2 | 3.8 | 6.1 | | 8.38E-02 | | 1 | | 0.8 | 2.4 | | 6.90E-01 | |
| Kininogen-1 | | | P01042 | 7 | 18.4 | 47.8 | 3 | 4.9 | 10.2 | 2.44E-01 | 2 | 2.9 | 6.4 | | 5.08E-01 | | 4 | | 6.0 | 8.8 | | 8.60E-01 | |
| Leucine-rich alpha-2-glycoprotein | | | P02750 | 7 | 25.8 | 47.6 | 5 | 47.5 | 123.8 | 8.70E-01 | 0 | 0.0 | 0.0 | | 6.12E-02 | | 3 | | 12.9 | 24.3 | | 4.89E-01 | |
| Neuroblast differentiation-associated protein AHNAK^†; †††; ††††^ | | | Q09666 | 9 | 14.0 | 24.8 | 3 | 2.1 | 5.1 | 2.91E-02 | 3 | 2.4 | 3.5 | | 2.62E-01 | | 0 | | 0.0 | 0.0 | | 5.22E-03 | |
| Obscurin^†; ††; ††††^ | | | Q5VST9 | 16 | 1767.1 | 3857.7 | 8 | 775.6 | 1967.0 | 3.00E-02 | 4 | 136.1 | 161.8 | | 1.50E-02 | | 9 | | 393.5 | 349.5 | | 2.92E-01 | |
| Opticin*^; †; ††; †††; ††††^ | | | Q9UBM4 | 16 | 163.7 | 95.1 | 9 | 111.6 | 294.7 | 7.39E-04 | 6 | 62.6 | 61.7 | | 1.83E-02 | | 8 | | 54.6 | 91.0 | | 1.87E-03 | |
| Osteopontin | | | P10451 | 13 | 53.1 | 80.0 | 11 | 85.1 | 184.3 | 7.54E-01 | 4 | 22.1 | 30.2 | | 3.74E-01 | | 10 | | 103.5 | 106.7 | | 7.29E-02 | |
| Pigment epithelium-derived factor^†; †††; ††††^ | | | P36955 | 15 | 161.2 | 134.1 | 14 | 145.8 | 56.7 | 9.01E-01 | 6 | 177.5 | 55.4 | | 3.38E-01 | | 10 | | 246.5 | 74.1 | | 1.77E-02 | |
| Plasminogen | | | P00747 | 7 | 12.8 | 18.6 | 3 | 3.2 | 6.5 | 1.19E-01 | 3 | 8.8 | 12.0 | | 9.04E-01 | | 3 | | 2.0 | 3.3 | | 2.66E-01 | |
| Prostaglandin-H2 D-isomerase | | | P41222 | 16 | 2589.7 | 2630.4 | 14 | 3005.7 | 3672.8 | 7.71E-01 | 5 | 1146.3 | 1044.4 | | 3.02E-01 | | 10 | | 2693.4 | 1025.5 | | 2.68E-01 | |
| Protein Jade-2 | | | Q9NQC1 | 2 | 0.2 | 0.7 | 1 | 0.2 | 0.8 | 6.90E-01 | 0 | 0.0 | 0.0 | | 3.75E-01 | | 0 | | 0.0 | 0.0 | | 2.54E-01 | |
| Protein S100-A9 | | | P06702 | 5 | 44.6 | 155.8 | 0 | 0.0 | 0.0 | 2.50E-02 | 1 | 0.2 | 0.6 | | 5.42E-01 | | 2 | | 0.4 | 1.0 | | 5.44E-01 | |
| Prothrombin | | | P00734 | 3 | 25.6 | 88.9 | 3 | 8.6 | 17.5 | 8.35E-01 | 4 | 42.8 | 58.4 | | 3.24E-02 | | 1 | | 6.7 | 21.0 | | 5.85E-01 | |
| Retinol-binding protein 3 | | | P10745 | 16 | 605.1 | 1113.3 | 14 | 343.7 | 753.6 | 4.60E-02 | 6 | 156.3 | 64.4 | | 2.10E-01 | | 10 | | 189.2 | 71.7 | | 4.29E-01 | |
| Serine/cysteine proteinase inhibitor clade G member 1 splice variant 2 | | | Q5UGI6 | 12 | 18.0 | 30.6 | 10 | 29.2 | 41.3 | 4.25E-01 | 3 | 7.0 | 11.4 | | 3.30E-01 | | 6 | | 17.7 | 24.7 | | 8.31E-01 | |
| Serotransferrin | | | P02787 | 16 | 323.7 | 181.1 | 14 | 229.8 | 68.7 | 3.83E-01 | 6 | 252.9 | 67.0 | | 5.07E-01 | | 10 | | 319.9 | 83.6 | | 5.62E-01 | |
| SERPINA3 Alpha-1-antichymotrypsin | | | P01011 | 16 | 43.1 | 30.3 | 14 | 46.8 | 27.0 | 5.61E-01 | 6 | 37.4 | 31.3 | | 5.07E-01 | | 10 | | 46.8 | 25.9 | | 8.74E-01 | |
| Serum albumin^†; ††; †††^ | | | P02768 | 16 | 2910.3 | 1163.1 | 14 | 1759.2 | 603.5 | 6.08E-03 | 6 | 2385.6 | 325.2 | | 2.69E-01 | | 10 | | 2434.6 | 670.3 | | 3.17E-01 | |
| Titin | | | Q8WZ42 | 6 | 3.5 | 6.1 | 7 | 7.1 | 9.2 | 3.58E-01 | 3 | 6.0 | 8.7 | | 5.63E-01 | | 6 | | 4.7 | 6.0 | | 3.90E-01 | |
| Translational activator GCN1^†; ††; ††††^ | | | Q92616 | 8 | 62.8 | 122.1 | 7 | 201.6 | 428.7 | 5.05E-01 | 4 | 367.8 | 316.7 | | 7.50E-02 | | 3 | | 51.7 | 103.9 | | 5.19E-01 | |
| Transthyretin | | | P02766 | 14 | 517.6 | 479.2 | 12 | 198.5 | 191.3 | 7.35E-02 | 6 | 306.7 | 177.1 | | 6.58E-01 | | 9 | | 368.1 | 328.6 | | 4.93E-01 | |
| Vitamin D-binding protein | | | **P02774** | 11 | 25.3 | 32.1 | 10 | 22.0 | 25.3 | 8.99E-01 | 6 | 39.1 | 41.4 | | 2.08E-01 | | 6 | | 11.9 | 16.2 | | 4.19E-01 | |
| Vitronectin*^; †; ††; †††; ††††^ | | | P04004 | 8 | 8.8 | 11.0 | 10 | 71.9 | 100.9 | 1.81E-02 | 6 | 38.3 | 16.0 | | 1.51E-03 | | 9 | | 145.1 | 197.6 | | 1.50E-03 | |
| Zinc-alpha-2-glycoprotein | | | **P25311** | 7 | 12.5 | 24.5 | 7 | 18.4 | 27.8 | 5.42E-01 | 4 | 17.5 | 20.8 | | 3.86E-01 | | 8 | | 76.9 | 78.2 | | 1.37E-02 | |
|  | | | | | | | | | | | | | | | | | | | | | | |  |
| ‡  #  §  †  * | Listed in the universal protein resource (UniProt), a central repository of protein data.  Number of samples with a signal intensity >0  *P*-Value was analyzed by using the Mann-Whitney test. A *P* of α<5.00E-02 was considered statistically significant. Significant results are highlighted in dark grey.  Closed testing procedure was used to verify the results. Kruskal-Wallis Test was used for analysis. A *P* of α<5.00E-02 was considered statistically significant. Proteins are marked when being significant in listed steps:  Step 1:  † Control vs. CRVO vs. H-CRVO vs. BRVO  Step 2:  †† Control vs. CRVO vs. H-CRVO  ††† Control vs. CRVO vs. BRVO  †††† Control vs. H-CRVO vs. BRVO  Step 3:  For detailed information of step 3 see §  Proteins which remained significant after performing multiple hypotheses testing correction, analyzed by using the Benjamini-Hochberg test for false discovery rate. | | | | | | | | | | | | | | | | | | | | | |  |
|  | Significant proteins in vitreous humor detected by CE-MS and identified by LC-MS/MS when comparing protein signal intensity of RVO-samples (n=30) compared to control-samples (n=16; see Tab. 2). | | | | | | | | | | | | | | | | | | | | | |  |
|  | |  | |  |  |  |  |  |  |  |  |  |  |  | |  | |  | |  |  | |  |
