# Supplementary material for: Proteomic Analysis of Vitreous Humor in Retinal Vein Occlusion
Source: PLoS One. 2016 Jun 30;11(6):e0158001. doi: 10.1371/journal.pone.0158001 (PMC4928959; doi:10.1371/journal.pone.0158001)
Supplement: S5 Table — (DOCX) [file pone.0158001.s006.docx]

| **Table S5. Influence of past time period since suffering retinal vein occlusion on biomarker discovery.**  Proteins in vitreous humor detected by capillary electrophoresis coupled to mass spectrometer (CE-MS) and identified by tandem mass spectrometry (LC-MS/MS) when comparing protein signal intensity of fresh/old retinal vein occlusion (RVO) compared to control-samples. | | | | | | | | | | | | | | |
| --- | --- | --- | --- | --- | --- | --- | --- | --- | --- | --- | --- | --- | --- | --- |
| **Protein** | | **UniPort^‡^** | **Control (n=16)** | | | **RVO-fresh (n=22)** | | | ***P*-Value^§^**  Control  vs.  RVO-fresh | **RVO-old (n=8)** | | | ***P* -Value^§^**  Control  vs.  RVO-old | ***P* -Value^§^**  RVO-fresh  vs.  RVO-old |
|  |  |  | N^#^ | Signal intensity | | N^#^ | Signal intensity | |  | N^#^ | Signal intensity | |  |  |
|  |  |  |  | Mean | SD |  | Mean | SD |  |  | Mean | SD |  |  |
| Afamin | | P43652 | 12 | 48.9 | 53.3 | 11 | 42.3 | 123.1 | 8.20E-02 | 8 | 13.3 | 4.6 | 2.20E-01 | 5.01E-01 |
| Alpha-1-acid glycoprotein 1 | | P02763 | 14 | 75.8 | 50.2 | 19 | 114.2 | 113.8 | 3.91E-01 | 8 | 227.1 | 139.7 | 2.69E-03 | 2.43E-02 |
| Alpha-1-acid glycoprotein 2 | | P19652 | 12 | 68.6 | 63.6 | 18 | 82.8 | 68.2 | 5.93E-01 | 8 | 97.7 | 75.4 | 3.90E-01 | 7.07E-01 |
| Alpha-1-antitrypsin | | P01009 | 16 | 272.4 | 101.1 | 22 | 283.8 | 166.1 | 5.95E-01 | 8 | 298.2 | 73.7 | 6.68E-01 | 3.99E-01 |
| Alpha-1B-glycoprotein | | P04217 | 4 | 5.7 | 14.7 | 5 | 12.0 | 31.4 | 9.37E-01 | 1 | 2.3 | 6.4 | 5.46E-01 | 4.81E-01 |
| Alpha-2-HS-glycoprotein | | P02765 | 8 | 10.3 | 16.5 | 6 | 5.1 | 11.1 | 1.51E-01 | 5 | 7.6 | 7.3 | 8.98E-01 | 1.15E-01 |
| Alpha-2-macroglobulin | | P01023 | 15 | 23.6 | 16.2 | 22 | 124.2 | 342.4 | 4.25E-01 | 8 | 24.7 | 13.1 | 5.40E-01 | 8.51E-01 |
| Alpha-crystallin B chain | | P02511 | 7 | 30.1 | 75.5 | 3 | 2.6 | 8.6 | 4.12E-02 | 0 | 0.0 | 0.0 | 3.28E-02 | 2.80E-01 |
| Amyloid-like protein 2 | | Q06481 | 9 | 5.6 | 9.4 | 9 | 10.9 | 31.6 | 6.43E-01 | 5 | 4.2 | 4.7 | 8.74E-01 | 5.58E-01 |
| Angiotensinogen | | P01019 | 8 | 49.8 | 83.5 | 8 | 18.0 | 38.1 | 1.99E-01 | 2 | 6.9 | 15.5 | 1.94E-01 | 5.02E-01 |
| Antithrombin-III | | P01008 | 14 | 50.2 | 31.7 | 20 | 69.1 | 36.8 | 1.47E-01 | 8 | 76.6 | 20.7 | 3.73E-02 | 6.06E-01 |
| Apolipoprotein A-I | | P02647 | 15 | 142.0 | 87.6 | 22 | 174.2 | 82.1 | 3.15E-01 | 8 | 212.7 | 48.4 | 3.73E-02 | 2.60E-01 |
| Apolipoprotein A-II | | P02652 | 10 | 50.1 | 67.6 | 5 | 3.9 | 8.2 | 6.00E-03 | 5 | 19.7 | 21.2 | 4.89E-01 | 1.75E-02 |
| Apolipoprotein A-IV | | P06727 | 16 | 147.4 | 352.9 | 22 | 45.0 | 39.7 | 5.54E-01 | 8 | 52.5 | 21.9 | 5.01E-01 | 3.25E-01 |
| Apolipoprotein E | | P02649 | 15 | 112.1 | 116.1 | 18 | 131.0 | 158.1 | 7.67E-01 | 8 | 101.1 | 121.8 | 6.24E-01 | 7.78E-01 |
| Beta-2-glycoprotein 1 | | P02749 | 4 | 4.6 | 12.8 | 2 | 0.3 | 1.1 | 1.36E-01 | 2 | 0.8 | 1.7 | 7.47E-01 | 2.67E-01 |
| Beta-crystallin B2 | | P43320 | 5 | 12.0 | 39.7 | 5 | 10.5 | 23.7 | 7.17E-01 | 6 | 39.8 | 52.5 | 6.16E-02 | 1.25E-02 |
| Calmodulin-regulated spectrin-associated protein 3 | | Q9P1Y5 | 3 | 1.6 | 4.9 | 2 | 58.2 | 192.2 | 4.81E-01 | 0 | 0.0 | 0.0 | 2.01E-01 | 3.86E-01 |
| Cartilage glycoprotein-39 | | Q9NY41 | 7 | 6.9 | 12.6 | 7 | 2.5 | 5.0 | 2.97E-01 | 5 | 4.5 | 4.4 | 5.78E-01 | 9.53E-02 |
| Cathepsin D | | P07339 | 15 | 553.9 | 1198.1 | 21 | 294.0 | 537.6 | 8.94E-01 | 8 | 99.2 | 74.6 | 5.40E-01 | 4.82E-01 |
| Ceruloplasmin | | P00450 | 15 | 157.1 | 187.6 | 22 | 229.3 | 203.9 | 8.64E-02 | 8 | 147.0 | 68.4 | 3.91E-01 | 4.82E-01 |
| Clusterin* | | P10909 | 15 | 282.3 | 174.6 | 22 | 551.2 | 209.2 | 6.04E-04 | 8 | 449.6 | 127.8 | 3.21E-02 | 2.41E-01 |
| Collagen alpha-1(I) chain | | P02452 | 4 | 5.1 | 11.3 | 10 | 6.2 | 8.3 | 3.05E-01 | 4 | 11.8 | 14.1 | 2.15E-01 | 4.15E-01 |
| Collagen alpha-1(II) chain | | P02458 | 15 | 371.1 | 1026.1 | 22 | 103.3 | 147.7 | 2.49E-01 | 8 | 74.5 | 82.6 | 7.13E-01 | 3.48E-01 |
| Collagen alpha-1(III) chain | | P02461 | 10 | 37.9 | 41.8 | 16 | 87.8 | 93.8 | 1.18E-01 | 7 | 108.6 | 89.4 | 3.50E-02 | 3.22E-01 |
| Collagen alpha-1(V) chain | | P20908 | 13 | 64.7 | 142.9 | 16 | 39.6 | 36.0 | 3.72E-01 | 6 | 23.1 | 38.7 | 3.89E-01 | 2.37E-01 |
| Collagen alpha-1(VII) chain | | Q02388 | 8 | 25.1 | 57.2 | 2 | 18.2 | 84.8 | 6.00E-03 | 3 | 59.7 | 140.4 | 8.41E-01 | 6.04E-02 |
| Collagen alpha-1(IX) chain | | P20849 | 12 | 73.1 | 187.1 | 14 | 21.5 | 36.9 | 9.76E-01 | 7 | 12.0 | 12.2 | 5.80E-01 | 9.62E-01 |
| Collagen alpha-1(XI) chain | | P12107 | 8 | 145.8 | 444.7 | 9 | 116.7 | 368.4 | 7.95E-01 | 5 | 9.8 | 17.8 | 5.62E-01 | 4.60E-01 |
| Collagen alpha-1(XII) chain | | Q99715 | 5 | 6.1 | 14.5 | 9 | 14.3 | 21.4 | 3.47E-01 | 5 | 10.8 | 11.6 | 1.42E-01 | 7.41E-01 |
| Collagen alpha-1(XXII) chain | | Q8NFW1 | 9 | 14.0 | 22.0 | 11 | 22.5 | 35.2 | 7.90E-01 | 7 | 28.3 | 38.9 | 2.01E-01 | 2.76E-01 |
| Collagen alpha-1(XXIV) chain | | Q17RW2 | 3 | 6.6 | 20.7 | 1 | 2.6 | 12.2 | 1.74E-01 | 0 | 0.0 | 0.0 | 2.01E-01 | 5.46E-01 |
| Collagen alpha-1(XXVII) chain | | Q8IZC6 | 2 | 257.3 | 854.5 | 3 | 1.4 | 4.4 | 9.60E-01 | 1 | 0.5 | 1.4 | 9.15E-01 | 9.05E-01 |
| Collagen alpha-1(XXVIII) chain | | Q2UY09 | 1 | 16.9 | 67.5 | 1 | 2.8 | 12.9 | 7.89E-01 | 0 | 0.0 | 0.0 | 4.80E-01 | 5.46E-01 |
| Collagen alpha-2(I) chain | | P08123 | 6 | 5.1 | 8.1 | 11 | 12.0 | 14.6 | 2.23E-01 | 7 | 23.4 | 16.7 | 3.76E-03 | 9.63E-02 |
| Collagen alpha-2(IX) chain | | Q14055 | 5 | 134.1 | 443.6 | 4 | 22.9 | 102.2 | 2.84E-01 | 2 | 1.5 | 3.4 | 5.94E-01 | 7.37E-01 |
| Collagen alpha-2(XI) chain | | P13942 | 13 | 377.1 | 528.5 | 22 | 815.0 | 681.6 | 7.12E-03 | 8 | 535.3 | 670.1 | 1.59E-01 | 8.27E-02 |
| Collagen alpha-3(IX) chain | | Q14050 | 11 | 155.8 | 156.3 | 13 | 71.5 | 116.4 | 1.33E-01 | 5 | 45.7 | 62.4 | 1.43E-01 | 9.81E-01 |
| Collagen alpha-4(IV) chain | | P53420 | 2 | 2.0 | 5.6 | 1 | 1.8 | 8.3 | 4.11E-01 | 0 | 0.0 | 0.0 | 3.07E-01 | 5.46E-01 |
| Collagen alpha-5(IV) chain | | P29400 | 16 | 264.0 | 353.1 | 18 | 251.6 | 272.7 | 9.06E-01 | 6 | 249.9 | 288.3 | 7.59E-01 | 8.51E-01 |
| Complement C3* | | P01024 | 16 | 390.2 | 194.5 | 22 | 594.1 | 132.0 | 1.91E-03 | 8 | 550.0 | 144.3 | 6.62E-02 | 4.25E-01 |
| Complement C4-A | | P0C0L4 | 7 | 29.3 | 78.3 | 3 | 1.1 | 2.8 | 2.82E-02 | 1 | 4.6 | 12.9 | 1.77E-01 | 9.68E-01 |
| Complement component 4B preproprotein | | P0C0L5 | 13 | 18.2 | 23.0 | 18 | 16.4 | 17.6 | 8.36E-01 | 8 | 22.5 | 15.3 | 2.44E-01 | 2.05E-01 |
| Complement component C9 | | P02748 | 13 | 245.2 | 194.7 | 21 | 296.3 | 182.9 | 6.25E-01 | 7 | 236.5 | 157.7 | 9.76E-01 | 3.60E-01 |
| Complement factor B | | P00751 | 11 | 44.9 | 61.4 | 7 | 19.6 | 69.6 | 8.32E-03 | 6 | 14.1 | 24.0 | 2.15E-01 | 1.47E-01 |
| Cystatin-C | | P01034 | 12 | 39.9 | 38.3 | 17 | 52.4 | 105.6 | 4.05E-01 | 8 | 37.3 | 23.9 | 8.06E-01 | 1.10E-01 |
| Dickkopf-related protein 3 | | Q9UBP4 | 14 | 129.9 | 142.7 | 19 | 93.0 | 111.3 | 3.00E-01 | 8 | 91.9 | 80.3 | 6.24E-01 | 7.43E-01 |
| Fibrinogen alpha chain | | P02671 | 6 | 12.0 | 31.1 | 9 | 6.7 | 10.2 | 8.93E-01 | 7 | 15.2 | 12.1 | 3.93E-02 | 4.51E-02 |
| Fibrinogen beta chain | | P02675 | 7 | 6.9 | 14.1 | 11 | 19.3 | 29.9 | 2.99E-01 | 7 | 49.4 | 51.3 | 5.70E-03 | 5.56E-02 |
| Fizzy-related protein homolog | | Q9UM11 | 6 | 2.7 | 4.4 | 6 | 4.7 | 10.8 | 7.47E-01 | 6 | 8.7 | 6.4 | 2.20E-02 | 5.01E-02 |
| Gelsolin | | P06396 | 12 | 29.5 | 66.1 | 11 | 26.0 | 49.9 | 5.62E-01 | 6 | 26.6 | 22.3 | 3.55E-01 | 2.61E-01 |
| Glutathione peroxidase 3 | | P22352 | 12 | 78.7 | 174.6 | 16 | 86.5 | 87.0 | 3.10E-01 | 8 | 93.6 | 79.0 | 9.75E-02 | 4.80E-01 |
| Haptoglobin | | P00738 | 10 | 30.6 | 51.3 | 16 | 39.4 | 94.5 | 6.31E-01 | 8 | 77.1 | 85.8 | 5.58E-02 | 9.01E-02 |
| Hemoglobin subunit beta | | P68871 | 7 | 38.6 | 136.9 | 9 | 117.6 | 454.9 | 5.87E-01 | 5 | 32.2 | 38.0 | 2.02E-01 | 4.01E-01 |
| Hemopexin | | P02790 | 15 | 159.3 | 92.7 | 22 | 127.4 | 59.4 | 1.47E-01 | 8 | 151.2 | 40.8 | 5.40E-01 | 2.05E-01 |
| Heparin cofactor 2 | | P05546 | 4 | 1.3 | 3.0 | 2 | 1.3 | 5.0 | 2.26E-01 | 2 | 1.3 | 2.7 | 1.00E+00 | 3.02E-01 |
| Histidine-rich glycoprotein | | P04196 | 4 | 6.9 | 17.7 | 4 | 10.6 | 31.0 | 7.40E-01 | 1 | 0.7 | 2.0 | 4.38E-01 | 6.13E-01 |
| Ig alpha-1 chain C region | | P01876 | 5 | 16.0 | 41.0 | 10 | 53.6 | 100.2 | 2.41E-01 | 3 | 5.6 | 8.5 | 8.55E-01 | 3.78E-01 |
| Ig alpha-2 chain C region | | P01877 | 11 | 84.2 | 88.9 | 17 | 143.7 | 165.4 | 3.03E-01 | 8 | 101.2 | 57.7 | 2.96E-01 | 8.88E-01 |
| Ig gamma-1 chain C region | | P01857 | 16 | 725.9 | 462.5 | 22 | 785.6 | 409.9 | 5.15E-01 | 8 | 793.7 | 170.6 | 4.26E-01 | 1.00E+00 |
| Ig gamma-2 chain C region | | P01859 | 14 | 65.0 | 94.3 | 14 | 39.3 | 47.1 | 3.71E-01 | 7 | 23.9 | 19.4 | 3.91E-01 | 9.62E-01 |
| Ig gamma-3 chain C region | | P01860 | 8 | 47.2 | 94.0 | 8 | 40.0 | 86.1 | 5.10E-01 | 4 | 57.6 | 97.3 | 8.44E-01 | 4.59E-01 |
| Ig heavy chain V-III region GAL | | P01781 | 7 | 40.3 | 65.3 | 5 | 6.2 | 18.8 | 8.85E-02 | 6 | 25.5 | 32.2 | 6.06E-01 | 1.07E-02 |
| Ig kappa chain C region | | P01834 | 11 | 110.8 | 108.3 | 19 | 119.3 | 117.5 | 9.17E-01 | 8 | 98.7 | 120.6 | 8.54E-01 | 4.82E-01 |
| Ig kappa chain V-I region EU | | P01598 | 3 | 3.1 | 7.4 | 3 | 8.5 | 34.3 | 6.92E-01 | 1 | 3.2 | 9.1 | 7.41E-01 | 9.68E-01 |
| Ig lambda-2 chain C regions | | P0CG05 | 12 | 452.7 | 305.4 | 18 | 765.7 | 584.2 | 9.04E-02 | 7 | 584.8 | 305.6 | 2.43E-01 | 4.52E-01 |
| IgGFc-binding protein | | Q9Y6R7 | 14 | 181.5 | 268.0 | 15 | 151.4 | 357.3 | 3.72E-01 | 5 | 23.1 | 23.0 | 6.50E-02 | 2.42E-01 |
| Ig lambda-like polypeptide 5* | | B9A064 | 11 | 93.4 | 75.7 | 22 | 336.4 | 372.9 | 1.52E-04 | 8 | 418.1 | 562.9 | 2.24E-04 | 9.25E-01 |
| Inter-alpha (Globulin) inhibitor H2 | | A2RTY6 | 6 | 12.5 | 26.5 | 12 | 16.8 | 22.3 | 3.06E-01 | 6 | 24.6 | 28.6 | 1.16E-01 | 3.32E-01 |
| Inter-alpha-trypsin inhibitor heavy chain H1 | | P19827 | 13 | 92.5 | 79.6 | 21 | 110.0 | 138.2 | 9.41E-01 | 7 | 102.2 | 104.6 | 9.76E-01 | 9.07E-01 |
| Inter-alpha-trypsin inhibitor heavy chain H4 | | Q14624 | 1 | 0.0 | 0.2 | 7 | 3.7 | 6.8 | 4.43E-02 | 1 | 5.6 | 15.7 | 5.66E-01 | 4.16E-01 |
| Kininogen-1 | | P01042 | 7 | 18.4 | 47.8 | 7 | 4.9 | 9.1 | 4.22E-01 | 2 | 4.8 | 9.1 | 4.39E-01 | 8.17E-01 |
| Leucine-rich alpha-2-glycoprotein | | P02750 | 7 | 25.8 | 47.6 | 5 | 30.4 | 100.2 | 2.59E-01 | 3 | 15.6 | 25.8 | 7.58E-01 | 5.67E-01 |
| Neuroblast differentiation-associated protein AHNAK | | Q09666 | 9 | 14.0 | 24.8 | 3 | 1.1 | 4.1 | 3.10E-03 | 3 | 2.3 | 3.3 | 1.24E-01 | 1.49E-01 |
| Obscurin | | Q5VST9 | 16 | 1767.1 | 3857.7 | 14 | 607.0 | 1582.5 | 2.05E-02 | 7 | 281.9 | 199.5 | 9.82E-02 | 6.69E-01 |
| Opticin* | | Q9UBM4 | 16 | 163.7 | 95.1 | 16 | 92.3 | 240.1 | 1.50E-04 | 7 | 56.6 | 61.4 | 7.05E-03 | 6.04E-01 |
| Osteopontin | | P10451 | 13 | 53.1 | 80.0 | 18 | 91.3 | 158.7 | 5.73E-01 | 7 | 44.0 | 68.2 | 7.82E-01 | 3.98E-01 |
| Pigment epithelium-derived factor | | P36955 | 15 | 161.2 | 134.1 | 22 | 185.9 | 82.5 | 2.25E-01 | 8 | 185.3 | 57.6 | 2.21E-01 | 7.78E-01 |
| Plasminogen | | P00747 | 7 | 12.8 | 18.6 | 6 | 3.4 | 7.3 | 1.52E-01 | 3 | 5.2 | 7.7 | 4.73E-01 | 4.52E-01 |
| Prostaglandin-H2 D-isomerase | | P41222 | 16 | 4122.9 | 6744.6 | 22 | 2990.7 | 2929.5 | 5.74E-01 | 7 | 1487.0 | 1012.4 | 2.45E-01 | 1.11E-01 |
| Protein Jade-2 | | Q9NQC1 | 2 | 0.2 | 0.7 | 0 | 0.0 | 0.0 | 9.28E-02 | 1 | 0.4 | 1.1 | 9.15E-01 | 9.73E-02 |
| Protein S100-A9 | | P06702 | 5 | 44.6 | 155.8 | 2 | 0.2 | 0.7 | 9.66E-02 | 1 | 0.1 | 0.4 | 3.14E-01 | 8.57E-01 |
| Prothrombin | | P00734 | 3 | 25.6 | 88.9 | 4 | 6.5 | 14.1 | 1.00E+00 | 4 | 37.7 | 54.8 | 1.09E-01 | 5.39E-02 |
| Retinol-binding protein 3 | | P10745 | 16 | 605.1 | 1113.3 | 22 | 278.7 | 600.8 | 4.44E-02 | 8 | 188.8 | 69.6 | 4.62E-01 | 3.25E-01 |
| Serine/cysteine proteinase inhibitor clade G member 1 splice variant 2 | | Q5UGI6 | 12 | 18.0 | 30.6 | 11 | 13.6 | 19.7 | 3.76E-01 | 8 | 41.2 | 50.5 | 5.71E-02 | 3.05E-02 |
| Serotransferrin | | P02787 | 16 | 323.7 | 181.1 | 22 | 264.5 | 88.7 | 6.36E-01 | 8 | 264.4 | 65.0 | 8.54E-01 | 8.51E-01 |
| SERPINA3 Alpha-1-antichymotrypsin | | P01011 | 16 | 43.1 | 30.3 | 22 | 42.9 | 27.7 | 8.59E-01 | 8 | 50.3 | 25.0 | 3.91E-01 | 4.53E-01 |
| Serum albumin | | P02768 | 16 | 2910.3 | 1163.1 | 22 | 2161.7 | 704.0 | 4.44E-02 | 8 | 1966.6 | 522.2 | 5.00E-02 | 3.73E-01 |
| Titin | | Q8WZ42 | 6 | 3.5 | 6.1 | 9 | 5.8 | 8.6 | 5.92E-01 | 7 | 6.8 | 6.3 | 9.41E-02 | 2.26E-01 |
| Translational activator GCN1 | | Q92616 | 8 | 62.8 | 122.1 | 9 | 185.1 | 385.0 | 8.46E-01 | 5 | 184.2 | 191.9 | 1.76E-01 | 3.46E-01 |
| Transthyretin | | P02766 | 14 | 517.6 | 479.2 | 19 | 242.4 | 252.7 | 1.04E-01 | 8 | 370.9 | 220.8 | 6.68E-01 | 1.11E-01 |
| Vitamin D-binding protein | | **P02774** | 11 | 25.3 | 32.1 | 14 | 14.6 | 24.3 | 5.07E-01 | 8 | 42.5 | 27.0 | 9.68E-02 | 4.50E-03 |
| Vitronectin* | | P04004 | 8 | 8.8 | 11.0 | 17 | 88.1 | 147.7 | 3.11E-03 | 8 | 93.7 | 105.4 | 2.96E-04 | 3.01E-01 |
| Zinc-alpha-2-glycoprotein | | **P25311** | 7 | 12.5 | 24.5 | 12 | 36.8 | 62.6 | 2.97E-01 | 7 | 40.2 | 33.9 | 2.03E-02 | 1.36E-01 |
|  | |  |  |  |  |  |  |  |  |  |  |  |  |  |
| ‡  #  §  * | Listed in the universal protein resource (UniProt), a central repository of protein data.  Number of samples with a signal intensity >0  *P*-Value was analyzed by using the Mann-Whitney test. A *P* of α<5.00E-02 was considered statistically significant. Significant results are highlighted in dark grey.  Proteins which remained significant after performing multiple hypotheses testing correction, analyzed by using the Benjamini-Hochberg test for false discovery rate. | | | | | | | | | | | | | |
|  | Significant proteins in vitreous humor detected by CE-MS and identified by LC-MS/MS when comparing protein signal intensity of RVO-samples (n=30) compared to control-samples (n=16; see Tab. 2). | | | | | | | | | | | | | |
|  | |  |  |  |  |  |  |  |  |  |  |  |  |  |
